# The Association Between Women's Perception of Birth During the Pandemic, Companion of Choice and Support From Health Professionals: A Cross‐Sectional Study in 20 Countries in the WHO European Region

**DOI:** 10.1111/birt.12915

**Published:** 2025-04-04

**Authors:** Stephanie Batram‐Zantvoort, Céline Miani, Ilaria Mariani, Emanuelle Pessa Valente, Mehreen Zaigham, Ingvild Hersoug Nedberg, Magdalena Kurbanović, Elizabete Pumpure, Anja Bohinec, Antigone Sarantaki, Barbara Baranowska, Alessia Abderhalden‐Zellweger, Elise de La Rochebrochard, Raquel Costa, Marina Ruxandra Otelea, Alina Liepinaitienė, Jelena Radetic, Amira Ćerimagić, Maryse Arendt, Martina König‐Bachmann, Stefano Delle Vedove, Karolina Linden, Sigrun Kongslien, Daniela Drandić, Darta Kreslina, Zalka Drglin, Dimitra Metallinou, Urszula Tataj‐Puzyna, Michael Gemperle, Virginie Rozée, Heloísa Dias, Marija Mizgaitienė, Jovana Ruzicic, Imola Simon, Simona Fumagalli, Helen Elden, Eline Skirnisdottir Vik, Barbara Mihevc Ponikvar, Aikaterini Lykeridou, Beata Szlendak, Claire de Labrusse, Tiago Miguel Pinto, Simona Jazdauskienė, Christoph Zenzmaier, Ilana Chertok, Emma Sacks, Marzia Lazzerini, IMAgiNE EURO Study group, Martina König‐Bachmann, Christoph Zenzmaier, Imola Simon, Elisabeth D’Costa, Anne Galle, Silke D’Hauwers, Amira Ćerimagić, Ourania Kolokotroni, Eleni Hadjigeorgiou, Maria Karanikola, Nicos Middleton, Ioli Orphanide Eteocleous, Daniela Drandić, Magdalena Kurbanović, Lenka Laubrova Zirovnicka, Miloslava Kramná, Virginie Rozée, Elise de La Rochebrochard, Kristina Löfgren, Céline Miani, Stephanie Batram‐Zantvoort, Antigoni Sarantaki, Dimitra Metallinou, Aikaterini Lykeridou, Ilana Chertok, Rada Artzi‐Medvedik, Marzia Lazzerini, Emanuelle Pessa Valente, Ilaria Mariani, Arianna Bomben, Stefano Delle Vedove, Sandra Morano, Antonella Nespoli, Simona Fumagalli, Elizabete Pumpure, Dace Rezeberga, Dārta Jakovicka, Gita Jansone‐Šantare, Anna Šibalova, Elīna Voitehoviča, Dārta Krēsliņa, Alina Liepinaitienė, Andželika Kondrakova, Marija Mizgaitienė, Simona Juciūtė, Maryse Arendt, Barbara Tasch, Enrico Lopriore, Thomas Van den Akker, Ingvild Hersoug Nedberg, Sigrun Kongslien, Eline Skirnisdottir Vik, Barbara Baranowska, Urszula Tataj‐Puzyna, Beata Szlendak, Paulina Pawlicka, Raquel Costa, Catarina Barata, Teresa Santos, Heloísa Dias, Tiago Miguel Pinto, Sofia Marques, Ana Meireles, Joana Oliveira, Mariana Pereira, Maria Arminda Nunes, Marina Ruxandra Otelea, Jelena Radetić, Jovana Ružičić, Zalka Drglin, Anja Bohinec, Serena Brigidi, Alejandra Oliden, Lara Martín Castañeda, Helen Elden, Karolina Linden, Mehreen Zaigham, Claire de Labrusse, Alessia Abderhalden‐Zellweger, Anouck Pfund, Harriet Thorn, Susanne Grylka, Michael Gemperle, Antonia Mueller, Emma Sacks

**Affiliations:** ^1^ Department of Epidemiology and International Public, School of Public Health Bielefeld University Bielefeld Germany; ^2^ Sexual and Reproductive Health and Rights Research Unit Institut National d'études démographiques (Ined) Aubervilliers Paris France; ^3^ WHO Collaborating Centre for Maternal and Child Health, Institute for Maternal and Child Health IRCCS Burlo Garofolo Trieste Italy; ^4^ Obstetrics & Gynecology Skåne University Hospital Malmö Sweden; ^5^ Department of Clinical Sciences Lund Lund University Lund Sweden; ^6^ Department of Health and Care Sciences UiT the Arctic University of Norway Harstad Norway; ^7^ Faculty of Health Studies University of Rijeka Rijeka Croatia; ^8^ Department of Obstetrics and Gynecology Riga Stradins University Riga Latvia; ^9^ Riga East Clinical University Hospital Riga Latvia; ^10^ National Institute of Public Health Ljubljana Slovenia; ^11^ Department of Midwifery University of West Attica Athens Greece; ^12^ Department of Midwifery Centre of Postgraduate Medical Education Warsaw Poland; ^13^ School of Health Sciences (HESAV) HES‐SO University of Applied Sciences and Arts Western Switzerland Lausanne Switzerland; ^14^ Laboratório Para a Investigação Integrativa e Translacional Em Saúde Populacional (ITR) Universidade do Porto Porto Portugal; ^15^ EPIUnit, ‐ Instituto de Saúde Pública Universidade do Porto Porto Portugal; ^16^ Lusófona University, HEI‐Lab: Digital Human‐Environment Interaction Laboratory, Universidade Lusófona Porto Portugal; ^17^ University of Medicine and Pharmacy Carol Davila Bucharest Romania; ^18^ Faculty of Natural Sciences, Department of Environmental Sciences Vytautas Magnus University Kaunas Lithuania; ^19^ Faculty of Medicine Kauno Kolegija Higher Education Institution Kaunas Lithuania; ^20^ Center for Moms Belgrade Serbia; ^21^ NGO Baby Steps Sarajevo Bosnia and Herzegovina; ^22^ Professional Association of Lactation Consultants Luxembourg; ^23^ Health University of Applied Sciences Tyrol Innsbruck Austria; ^24^ Institute of Health and Care Sciences, Sahlgrenska Academy, University of Gothenburg Gothenburg Sweden; ^25^ Roda ‐ Parents in Action Zagreb Croatia; ^26^ Institute of Midwifery and Reproductive Health, School of Health Sciences ZHAW Zurich University of Applied Sciences Winterthur Switzerland; ^27^ Université Paris‐Saclay, UVSQ, Inserm, CESP Villejuif France; ^28^ Regional Health Administration of the Algarve (ARS Algarve, IP) Faro Portugal; ^29^ Christian Maternity Unit, Lithuanian Hospital of Health Sciences Kaunas Lithuania; ^30^ Health Department University of Applied Sciences Burgenland Pinkafeld Austria; ^31^ School of Medicine and Surgery University of Milano‐Bicocca Monza Italy; ^32^ Department of health and caring sciences Western Norway University of Applied Sciences Norway; ^33^ Department of Obstetrics and Gynaecology Hospital of Lithuanian University of Health Sciences Kauno Klinikos Kaunas Lithuania; ^34^ Ohio University College of Health Sciences and Professions Athens USA; ^35^ Department of International Health Johns Hopkins School of Public Health Baltimore Maryland USA; ^36^ Maternal Adolescent Reproductive and Child Health Care Centre, Faculty of Epidemiology and Population Health, London School of Hygiene & Tropical Medicine London UK

**Keywords:** companion of choice, COVID‐19, disrespect and abuse, maternal experiences, positive birth perception, respectful maternity care, WHO standards

## Abstract

**Background:**

Mitigation measures implemented in response to the COVID‐19 pandemic led to significant changes in maternity care across Europe, including restrictions on companions during labor and birth. This cross‐sectional study explores the association between the presence of a companion of choice and a positive perception of the birth experience. Additionally, it explores the association between health professionals' attention, assistance, and availability during labor and birth and a positive perception of birth.

**Methods:**

We utilized a structured, validated online questionnaire, available in 25 languages, to assess the quality of maternal care during the COVID‐19 pandemic from women's perspectives. We conducted logistic regression to explore associations between variables related to the presence of a companion of choice, health professionals' attention, assistance, and availability, and positive perceptions of birth, when controlled for confounders, including birth mode and medical interventions.

**Results:**

Responses from 48,039 women across 20 countries in the WHO European Region were included. Always having a companion of choice during birth (aOR: 2.11) and always receiving adequate care from health professionals (assistance aOR: 2.12, attention aOR: 36.64, availability aOR: 2.12) were associated with positive birth perception. Instrumental births (aOR: 0.76), episiotomies (aOR: 0.74), fundal pressure (aOR: 0.52), and cesarean births (planned aOR: 0.80, unplanned prelabor aOR: 0.60, unplanned in‐labor aOR: 0.52) were associated with less positive birth perceptions.

**Discussion:**

This study highlights the critical role of having a chosen companion and receiving adequate attention, assistance, and availability from health professionals in promoting positive birth perceptions, even in times of crisis such as the COVID‐19 pandemic. Ensuring the presence of a companion of choice and comprehensive professional support is crucial for delivering high‐quality, respectful maternity care.

## Graphical abstract

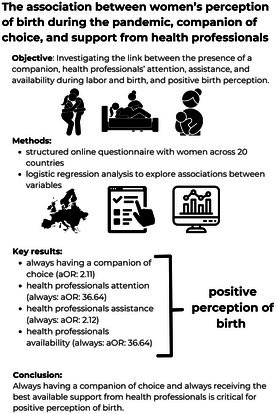

## 1 Background

During the COVID‐19 pandemic, policymakers and health authorities in European countries imposed restrictions on access to maternity care facilities for birth companions and visitors, as a measure to slow the spread of the virus [1, 2]. These bans were implemented despite the evidence that shows the benefit of having a birth companion on health outcomes and a positive childbirth experience [3, 4]. The restrictions varied over time, among countries, and across facilities [5]. In some hospitals, birth companions were permitted under strict regulations, such as mandatory real‐time polymerase chain reaction (RT‐PCR) testing and mask‐wearing. Others allowed companions only during the second phase of labor. In certain countries, access to the maternity ward was restricted to patients, meaning that women (We include into our considerations all pregnant and childbearing individuals.) had to navigate labor, birth, and the postpartum period without a birth companion [2, 6]. International [7] and national (e.g., Germany [8], Italy [9], France [10]) obstetricians' and midwives' associations, and civil society initiatives voiced concerns and formally opposed bans on birth companions. These initiatives referenced the World Health Organization's (WHO) recommendation to ensure women have a companion of choice during birth, emphasizing it as a vital element of respectful and high‐quality maternity care and a fundamental right in childbirth [3, 4]. Restrictions on companionship can evoke concerns, psychological distress, and loneliness for women in labor [11], placing added strain on their partner or desired companion [12], the newborn [13], and health professionals providing care [14]. Having a birth companion of choice, whether the woman's partner, a relative, doula, or a friend, can positively influence the labor and birth process, as companions can support and empower the woman [15], offer emotional support, practical assistance during labor, serve as an informational and communicational bridge between health professionals and the woman, and advocate for the woman's wishes. The significance and advantages of having a birth companion of choice are well‐documented [16, 17].

An equally important aspect of respectful and high‐quality maternity care is the continuous support and care provided by a health professional (usually a midwife) during labor and birth [18, 19]. Health professionals can enhance outcomes through evidence‐based care and supportive actions, including comfort, emotional support, effective communication, and information sharing, thereby mirroring the benefits of a birth companion of choice [20, 21]. Inadequate ratios of health professionals to women can lead to stressful work environments and unnecessary medical interventions during labor and can negatively impact women's birth experiences [22]. It also compromises a woman's right to receive the highest attainable level of health care during childbirth [4]. During the pandemic, inadequate staffing in maternity wards intensified due to health professionals' own illness quarantine, reduced working hours due to stressful working conditions, parental and caregiving duties, or redistribution of maternity staff to COVID‐19 wards [23]. Inadequate ratios of staff to women also increases the chances that women will experience mistreatment, disrespect or abuse during labor and birth [24, 25].

We examined data from 20 countries to determine whether having a birth companion of choice and receiving timely and sufficient attendance by an adequate number of health professionals during labor and birth are associated with positive birth perception when controlling for potential confounders. Positive birth perception refers to a woman's assessment of how she is being valued and treated as an individual, including preserving her integrity and autonomy [26, 27].

## 2 Methods

### 2.1 Study Design, Participants, and Data Collection

This study is part of the ongoing IMAgiNE EURO (Improving MAternal Newborn CarE In the EURO Region) project, which is collecting cross‐sectional data women's perspectives of the quality of maternal and newborn care (QMNC) in health facilities. Eligible participants included women aged 18 or older who gave birth in a maternity facility in the WHO European Region during the COVID‐19 pandemic (data collection start: March 1, 2020, data download for this study: March 1, 2023). Participants had the option of reporting on multiple births if they occurred within the study period. A structured, validated online questionnaire [28] was disseminated by project partners from over 20 countries and in 25 languages. Questions were based on WHO Quality Measures for improving the QMNC in health facilities, [29] using the three WHO domains of care (provision of care, experience of care, and availability of human and physical resources). Other questions pertained to organizational changes related to the COVID‐19 pandemic and sociodemographic data. The questionnaire design and setting‐specific dissemination strategies were described in prior publications [28, 30, 31, 32, 33, 34]. The Strengthening the Reporting of Observational Studies in Epidemiology (STROBE) guidelines for reporting on cross‐sectional studies were applied [35] (Supporting Information: File S1).

### 2.2 Study Variables

#### 2.2.1 Outcome Variable: Positive Birth Perception

Positive birth perception is defined as a woman's assessment of how she is valued and treated as an individual, and includes the preservation of her integrity and autonomy [26, 27, 36]. This was assessed using five key variables, which women then assessed on a scale of always/sometimes/never. Women reported on these five variables were as follows: (i) feeling treated with dignity, (ii) feeling emotionally supported by health professionals, (iii) feeling fully involved in choices regarding care and feeling that autonomy was respected, (iv) perceiving health professionals' communication to be clear and effective, and (v) reporting any kind of mistreatment, disrespect, or abuse (verbal, emotional, and physical). Using these five variables, we calculated a composite discrete score of positive birth perception ranging from 0 to 10, with 10 being the most positive perception of birth and 0 being the least positive perception of birth (with the following scoring for variables i–iv: always = 2 points, sometimes = 1 point, never = 0 points, and the following scoring for variable v: never = 2 points, sometimes = 1 point, always = 0). We dichotomized the birth perception score (10/< 10). A score equal to 10 indicates fully positive perception of birth. Due to the distribution of the variables, it seemed reasonable to dichotomize between (10/< 10). While recognizing that scores of 9, 8, or 7 might still be considered positive perceptions of birth, our aim with this study is to better understand the factors that contribute to a fully positive perception of birth.

#### 2.2.2 Independent Variables

Independent variables were measured using the same scale as outcome variables (always/sometimes/never). Having a birth companion of choice was measured through a variable that asked if participants were allowed to have their chosen companion present during and after birth. Health professionals' attention, assistance, and availability during labor and birth from the participants' perspective was measured through three variables: (i) whether the number of health professionals was adequate considering the workload; (ii) whether the health professionals were present in sufficient numbers to guarantee adequate assistance; and (iii) whether participants felt they received immediate attention from health professionals when they needed it.

#### 2.2.3 Potential Confounders

Socioeconomic and health‐related variables considered potentially relevant to the birth process and the provision of maternity care include maternal age, education, country, whether the mother was born in the same country where she gave birth to the infant, mode of birth, and birth interventions [37, 38, 39].

#### 2.2.4 Data Analysis

Responses missing ≥ 90% of key variables, suspected duplicates, and entries with missing values in independent or outcome variables were excluded from the dataset, as described elsewhere [28]. Descriptive statistics were used to explore the distribution of outcomes and independent variables. Considering the skewed score distribution, we displayed the positive birth perception score distribution from 0 to 10 overall and for all countries graphically. We performed chi‐squared tests to assess whether independent variables differed by the dichotomized positive birth perception score. Lastly, we conducted a multivariate logistic regression analysis to estimate the associations between the dichotomized outcome of positive birth perception (outcome variable), presence of a birth companion of choice, and health professionals' attention and assistance (three variables). We controlled for the following potential confounders: age, education, parity, country, whether the mother was born in the same country where she gave birth, birth mode, and presence of medical interventions (vaginal birth, vaginal birth with episiotomy, instrumental vaginal birth, instrumental vaginal birth with fundal pressure, planned cesarean birth, and unplanned cesarean birth before or after the onset of labor). The category with higher frequency was chosen as the reference. For the sensitivity analysis, we performed a linear regression model on a subset of women with a score < 10 to explore associations between not‐perfect scores and the variables of interest. The discrete score was used as an outcome variable, while we used the same independent variables used in the logistic model. Statistical analyses were performed using Stata/SE version 14.0 (Stata Corporation, College Station, TX, USA) and R version 4.2.2: A language and environment for statistical computing (R Foundation for Statistical Computing, Vienna, Austria. URL https://www.R‐project.org/).

### 2.3 Ethics Aspects

This voluntary and anonymous study was approved by the Institutional Review Board of the IRCCS Burlo Garofolo Trieste (IRB‐ Burlo 05/2020 15.07.2020) (Coordination center). The study was conducted according to General Data Protection Regulation (GDPR) regulations. Before participation, participants were informed of the objectives and methods of the study, including their rights in declining participation. Consent was provided before responding to the questionnaire, and participants could stop responding at any time. The study was approved by the ethics committees in four partner countries: Portugal (Instituto de Saúde Pública da Universidade do Porto, CE20159); Norway (Norwegian Regional Committee for Medical Research Ethics, 2020/213047), Germany (Bielefeld University ethics committee, 2020‐176), and Latvia (Rīgas Stradiņa universitātes, 22‐2/140/2021‐16/03/2021). Ethics approval was not necessary in other partner countries, as anonymity in data collection during the survey phase was ensured and no data were collected that disclosed participants' identities. Data transmission and storage were secured by encryption.

## 3 Results

### 3.1 Sample Characteristics

**FIGURE 1:**
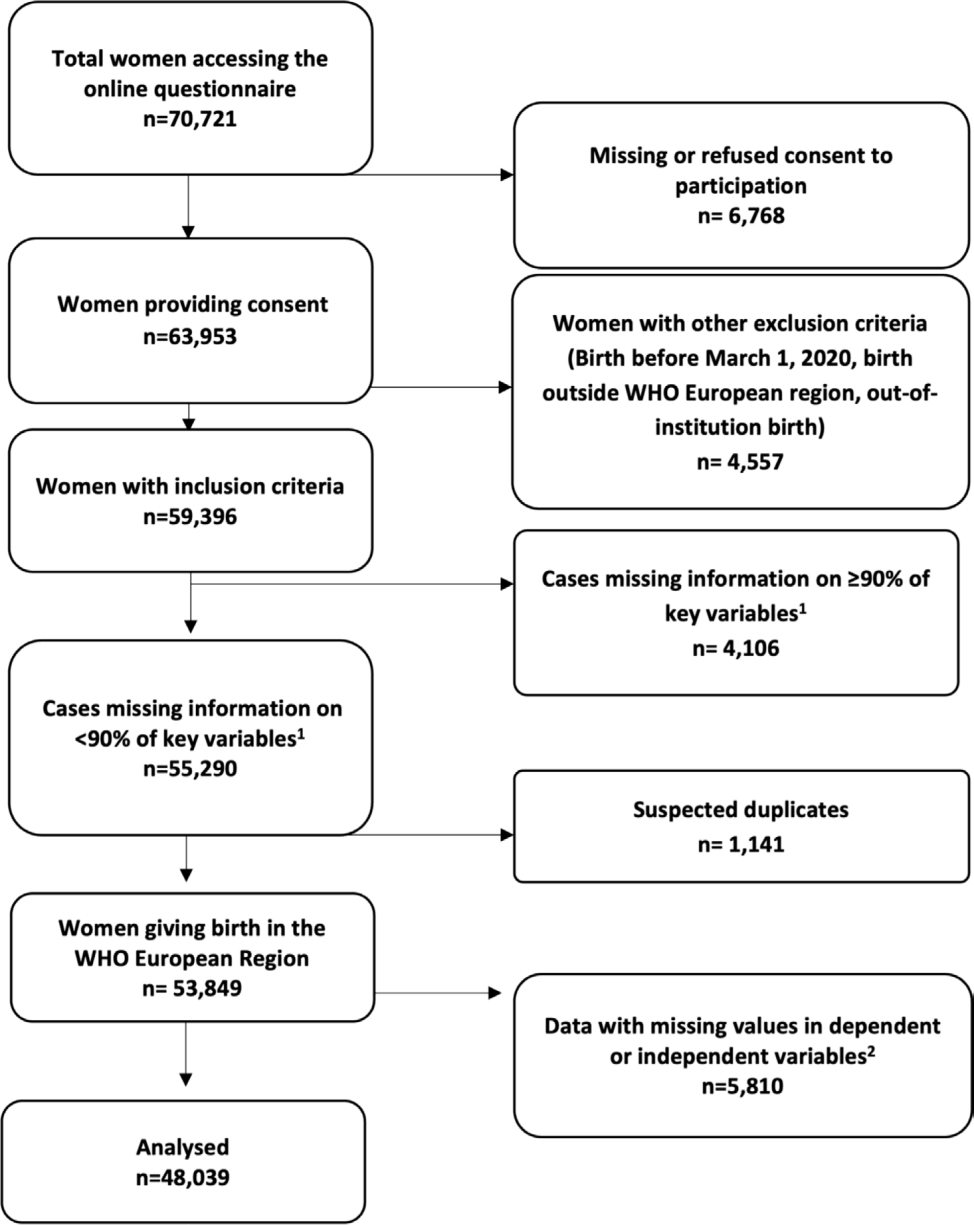
Flow diagram. ^1^We used 45 key variables (40 key quality measures and five key sociodemographic questions). ^2^Independent variables are as follows: dignity, emotional support, involvement, abuse, and communication. Dependent variables are as follows: companion of choice, adequate assistance by a health professional, adequate number of health professionals, and immediate attention by a health professional.

A total of 70,721 participants accessed the online questionnaire, from which 48,039 matched the inclusion criteria for this study (Figure 1).

The study sample characteristics are summarized in Table 1. More than half of the participants were highly educated (over 70.0% (*n* = 34,416) having a university degree), between 25 and 35 years old (74.1%; *n* = 35,808), and reported on their first birth (60.9%; *n* = 29,275). Most births were attended by a midwife (88.9%; *n* = 42,700) and/or an obstetrician, medical doctor, or medical resident (62.6%, *n* = 30,073). Over 90% (*n* = 44,445) of women gave birth in the same country where they were born.

**TABLE 1:** Characteristics of the sample.

	Overall *N* = 48,039
	*n* (%)
Parity	
Primiparous	29,275 (60.9%)
Multiparous	18,764 (39.1%)
Health professional(s) present	
Midwife	42,700 (88.9%)
Nurse	18,071 (37.6%)
Student	6945 (14.5%)
Obstetrician, medical doctor, or medical resident	30,073 (62.6%)
Woman gave birth in the same country where she was born	
Yes	44,445 (92.5%)
No	3594 (7.5%)
Educational level	
Junior high school or lower	2589 (5.4%)
High school	11,034 (23%)
University degree	19,531 (40.7%)
Postgraduate/master/doctorate	14,885 (31%)
Age	
18–24	2389 (5%)
25–30	16,544 (34.4%)
31–35	19,264 (40.1%)
36–39	7562 (15.7%)
> 40	2280 (4.7%)
Country	
Austria	406 (0.8%)
Bosnia‐Herzegovina	560 (1.2%)
Croatia	3241 (6.7%)
France	1410 (2.9%)
Greece	2214 (4.6%)
Germany	1300 (2.7%)
Italy	10,130 (21.1%)
Latvia	3220 (6.7%)
Lithuania	1196 (2.5%)
Luxembourg	515 (1.1%)
Norway	5441 (11.3%)
Poland	1866 (3.9%)
Portugal	1400 (2.9%)
Romania	1265 (2.6%)
Serbia	1102 (2.3%)
Slovenia	2558 (5.3%)
Spain	355 (0.7%)
Sweden	7729 (16.1%)
Switzerland	1416 (2.9%)
Other^a^	715 (1.5%)

^a^ Other countries include Albania, Armenia, Belgium, Bulgaria, Cyprus, Czech Republic, Denmark, Estonia, Finland, Hungary, Iceland, Ireland, Israel, Macedonia, Malta, Montenegro, Netherlands, Republic of Moldova, Slovakia, Turkey, and Ukraine.

As displayed in Table 2, 74.0% (*n* = 35,491) of all women gave birth vaginally, 49.7% (23,863) had no medical interventions (as defined in this study) during their vaginal birth, whereas 16.2% (*n* = 7781) gave birth vaginally with an episiotomy. Another 4.5% (*n* = 2141) of all women experienced an instrumental vaginal birth, and a further 3.6% (*n* = 1706) experienced instrumental vaginal birth with fundal pressure. In all, 26% (*n* = 12,548) of women had a cesarean birth. Of the total sample, planned cesarean births were reported by 10.8% (*n* = 5192), 5.2% (*n* = 2475) reported an unplanned cesarean birth before the onset of labor, and 10.2% (*n* = 4881) reported an unplanned cesarean birth after the onset of labor.

**TABLE 2:** Descriptive statistics on indicators of positive perception of birth.

	Overall	Dignified care			Emotional support			Involvement in decision‐making			Abuse			Effective communication		
		Always	Sometimes	Never	Always	Sometimes	Never	Always	Sometimes	Never	Always	Sometimes	Never	Always	Sometimes	Never
	*N* = 48,039	*N* = 35,089	*N* = 10,614	*N* = 2336	*N* = 32,972	*N* = 9918	*N* = 5149	*N* = 28,668	*N* = 12,874	*N* = 6497	*N* = 1247	*N* = 5964	*N* = 40,828	*N* = 32,108	*N* = 12,962	*N* = 2969
	*n* (%)	*n* (%)	*n* (%)	*n* (%)	*n* (%)	*n* (%)	*n* (%)	*n* (%)	*n* (%)	*n* (%)	*n* (%)	*n* (%)	*n* (%)	*n* (%)	*n* (%)	*n* (%)
By mode of birth and medical interventions^a^																
Vaginal birth	23,863 (49.7%)	18,932 (54.0%)	4221 (39.8%)	710 (30.4%)	18,154 (55.1%)	4084 (41.2%)	1625 (31.6%)	16,136 (56.3%)	5560 (43.2%)	2167 (33.4%)	415 (33.3%)	2198 (36.9%)	21,250 (52.0%)	17,373 (54.1%)	5464 (42.2%)	1026 (34.6%)
Vaginal birth with episiotomy	7781 (16.2%)	5210 (14.8%)	2029 (19.1%)	542 (23.2%)	4783 (14.5%)	1852 (18.7%)	1146 (22.3%)	3905 (13.6%)	2364 (18.4%)	1512 (23.3%)	276 (22.1%)	1193 (20.0%)	6312 (15.5%)	4690 (14.6%)	2425 (18.7%)	666 (22.4%)
Instrumental vaginal birth	2141 (4.5%)	1574 (4.5%)	476 (4.5%)	91 (3.9%)	1490 (4.5%)	470 (4.7%)	181 (3.5%)	1300 (4.5%)	615 (4.8%)	226 (3.5%)	59 (4.7%)	288 (4.8%)	1794 (4.4%)	1411 (4.4%)	608 (4.7%)	122 (4.1%)
Instrumental vaginal birth with fundal pressure	1706 (3.6%)	1046 (3.0%)	510 (4.8%)	150 (6.4%)	932 (2.8%)	455 (4.6%)	319 (6.2%)	802 (2.8%)	585 (4.5%)	319 (4.9%)	88 (7.1%)	336 (5.6%)	1282 (3.1%)	983 (3.1%)	571 (4.4%)	152 (5.1%)
Planned cesarean birth	5192 (10.8%)	3722 (10.6%)	1210 (11.4%)	260 (11.1%)	3592 (10.9%)	1049 (10.6%)	551 (10.7%)	2951 (10.3%)	1394 (10.8%)	847 (13.0%)	112 (9.0%)	604 (10.1%)	4476 (11.0%)	3406 (10.6%)	1468 (11.3%)	318 (10.7%)
Unplanned cesarean birth before the onset of labor	2475 (5.2%)	1653 (4.7%)	658 (6.2%)	164 (7.0%)	1488 (4.5%)	599 (6.0%)	388 (7.5%)	1220 (4.3%)	743 (5.8%)	512 (7.9%)	75 (6.0%)	371 (6.2%)	2029 (5.0%)	1492 (4.6%)	782 (6.0%)	201 (6.8%)
Unplanned cesarean birth, after the onset of labor	4881 (10.2%)	2952 (8.4%)	1510 (14.2%)	419 (17.9%)	2533 (7.7%)	1409 (14.2%)	939 (18.2%)	2354 (8.2%)	1613 (12.5%)	914 (14.1%)	222 (17.8%)	974 (16.3%)	3685 (9.0%)	2753 (8.6%)	1644 (12.7%)	484 (16.3%)
Immediate Attention from health professional																
Always	32,295 (67.2%)	28,865 (82.3%)	3275 (30.9%)	155 (6.6%)	26,217 (79.5%)	4927 (49.7%)	1151 (22.4%)	24,482 (85.4%)	6275 (48.7%)	1538 (23.7%)	242 (19.4%)	1849 (31%)	30,204 (74%)	28,107 (87.5%)	4031 (31.1%)	157 (5.3%)
Sometimes	12,721 (26.5%)	5819 (16.6%)	5961 (56.2%)	941 (40.3%)	5969 (18.1%)	4192 (42.3%)	2560 (49.7%)	3902 (13.6%)	5662 (44%)	3157 (48.6%)	465 (37.3%)	2970 (49.8%)	9286 (22.7%)	3783 (11.8%)	7705 (59.4%)	1233 (41.5%)
Never	3023 (6.3%)	405 (1.2%)	1378 (13.0%)	1240 (53.1%)	786 (2.4%)	799 (8.1%)	1438 (27.9%)	284 (1.0%)	937 (7.3%)	1802 (27.7%)	540 (43.3%)	1145 (19.2%)	1338 (3.3%)	218 (0.7%)	1226 (9.5%)	1579 (53.2%)
Birth companion of choice																
Always	19,988 (41.6%)	17,338 (49.4%)	2399 (22.6%)	251 (10.7%)	16,072 (48.7%)	3008 (30.3%)	908 (17.6%)	14,364 (50.1%)	4353 (33.8%)	1271 (19.6%)	285 (22.9%)	1515 (25.4%)	18,188 (44.5%)	15,688 (48.9%)	3796 (29.3%)	504 (17.0%)
Sometimes	7341 (15.3%)	5650 (16.1%)	1464 (13.8%)	227 (9.7%)	5293 (16.1%)	1481 (14.9%)	567 (11.0%)	4673 (16.3%)	1941 (15.1%)	727 (11.2%)	145 (11.6%)	841 (14.1%)	6355 (15.6%)	5176 (16.1%)	1821 (14.0%)	344 (11.6%)
Never	20,710 (43.1%)	12,101 (34.5%)	6751 (63.6%)	1858 (79.5%)	11,607 (35.2%)	5429 (54.7%)	3674 (71.4%)	9631 (33.6%)	6580 (51.1%)	4499 (69.2%)	817 (65.5%)	3608 (60.5%)	16,285 (39.9%)	11,244 (35.0%)	7345 (56.7%)	2121 (71.4%)
Adequate assistance by health professional																
Always	19,861 (41.3%)	17,969 (51.2%)	1737 (16.4%)	155 (6.6%)	16,593 (50.3%)	2570 (25.9%)	698 (13.6%)	15,586 (54.4%)	3364 (26.1%)	911 (14.0%)	205 (16.4%)	1100 (18.4%)	18,556 (45.4%)	17,266 (53.8%)	2387 (18.4%)	208 (7.0%)
Sometimes	17,618 (36.7%)	12,251 (34.9%)	4646 (43.8%)	721 (30.9%)	11,157 (33.8%)	4358 (43.9%)	2103 (40.8%)	9438 (32.9%)	5628 (43.7%)	2552 (39.3%)	415 (33.3%)	2386 (40.0%)	14,817 (36.3%)	10,992 (34.2%)	5723 (44.2%)	903 (30.4%)
Never	10,560 (22.0%)	4869 (13.9%)	4231 (39.9%)	1460 (62.5%)	5222 (15.8%)	2990 (30.1%)	2348 (45.6%)	3644 (12.7%)	3882 (30.2%)	3034 (46.7%)	627 (50.3%)	2478 (41.5%)	7455 (18.3%)	3850 (12.0%)	4852 (37.4%)	1858 (62.6%)
Adequate number of health professionals																
Always	31,808 (66.2%)	27,038 (77.1%)	4309 (40.6%)	461 (19.7%)	24,789 (75.2%)	5252 (53.0%)	1767 (34.3%)	22,751 (79.4%)	6779 (52.7%)	2278 (35.1%)	383 (30.7%)	2419 (40.6%)	29,006 (71.0%)	25,515 (79.5%)	5683 (43.8%)	610 (20.5%)
Sometimes	12,088 (25.2%)	6744 (19.2%)	4420 (41.6%)	924 (39.6%)	6526 (19.8%)	3487 (35.2%)	2075 (40.3%)	4926 (17.2%)	4593 (35.7%)	2569 (39.5%)	439 (35.2%)	2377 (39.9%)	9272 (22.7%)	5580 (17.4%)	5322 (41.1%)	1186 (39.9%)
Never	4143 (8.6%)	1307 (3.7%)	1885 (17.8%)	951 (40.7%)	1657 (5.0%)	1179 (11.9%)	1307 (25.4%)	991 (3.5%)	1502 (11.7%)	1650 (25.4%)	425 (34.1%)	1168 (19.6%)	2550 (6.2%)	1013 (3.2%)	1957 (15.1%)	1173 (39.5%)

^a^ Medical interventions: vaginal birth with episiotomy, instrumental vaginal birth, instrumental vaginal birth with fundal pressure, planned cesarean birth, unplanned cesarean birth before or after the onset of labor.

Overall, Table 2 shows that 41.6% (*n* = 19,988) of respondents reported that having a birth companion was always possible, 15.3% (*n* = 7341) reported sometimes, and 43.1% (*n* = 20,710) reported never. Over two‐thirds of the women felt that they always received immediate attention when needed (67.2%; *n* = 32,295) and that health professionals were available in adequate numbers all the time (always: 66.2%; *n* = 31,808). Overall, 41.3% (*n* = 19,861) respondents reported that they always experienced adequate assistance by health professionals.

Descriptive statistics for indicators of positive birth perception reveal that 48.7% (*n* = 16,072) of women who were always accompanied by a companion always felt emotionally supported, 50.1% (*n* = 14,364) always felt involved in decision‐making, 48.9% (*n* = 15,688) always experienced clear and effective communication from health professionals, 49.4% (*n* = 17,338) always felt treated with dignity, and 44.5% (*n* = 18,188) never experienced abuse. Conversely, among women who reported never receiving immediate attention from their health professional, 53.1% (*n* = 1240) reported never being treated with dignity, 27.9% (*n* = 1438), never feeling supported, 27.7% (*n* = 1802) reported never being involved in decision‐making, and 53.2% (*n* = 1579) reported never having effective communication. Of this same group, 43.3% (*n* = 540) reported always experiencing abuse. Generally, the measures for immediate attention, adequate assistance, and an adequate number of health professionals show similar distributions in the positive perception of birth indicators, as shown in Table 2.

### 3.2 Positive Perception of Birth

Around 43% (*n* = 20,918) of all women reported a positive birth perception score of 10, with the remaining scores ranging from 9 to 0, with decreasing frequency as the score decreases. There was a variation in score distribution across countries. Distribution for individual countries and overall can be found in Supporting Information: File S2.

### 3.3 Multivariate Analyses

As displayed in Table 3, the logistic regression model shows that reporting being always (aOR: 2.11; CI: 2.00; 2.23) or at least sometimes (aOR: 1.45; CI: 1.35; 1.55) having a birth companion of choice was associated with increased odds of a positive birth experience compared to women who did not have a birth companion of choice. All variables on health professionals' availability, assistance, and attention also showed a significant association with a positive birth perception. Reporting being always adequately assisted throughout labor and birth (aOR: 2.12; CI: 1.86; 2.43), always receiving immediate attention (aOR: 36.64; CI: 27.77; 49.55), and reporting that there was always an adequate number of health professionals available (aOR: 2.12; CI: 1.95; 2.31) were all associated with higher odds of a positive perception of birth. Having health professionals available sometimes (aOR: 1.10; CI: 1.02; 1.19) and receiving their assistance (aOR: 1.24; CI: 1.09; 1.41) and attention sometimes (aOR: 4.91; CI: 3.71; 6.66) were also associated with a higher probability of a positive perception of birth compared with women who reported never receiving assistance or attention by health professionals or who reported that health professionals were not available in adequate numbers.

**TABLE 3:** Logistic model result, positive birth perception is dichotomized 10 versus < 10 (*n* = 48,039).

Logistic model			
Positive birth perception (composite outcome)			
	Odds ratio	95% CI	*p*‐value
Companion of choice allowed			
Always	2.11	2; 2.23	< 0.001
Sometimes	1.45	1.35; 1.55	< 0.001
Never	Ref	Ref	
Adequate number of health professionals			
Always	2.12	1.95; 2.31	< 0.001
Sometimes	1.1	1.02; 1.19	0.013
Never	Ref	Ref	
Adequate assistance by health professional			
Always	2.12	1.86; 2.43	< 0.001
Sometimes	1.24	1.09; 1.41	< 0.001
Never	Ref	Ref	
Immediate attention by health professional			
Always	36.64	27.77; 49.55	< 0.001
Sometimes	4.91	3.71; 6.66	< 0.001
Never	Ref	Ref	
By birth mode and medical interventions^a^			
Vaginal birth	Ref	Ref	
Vaginal birth with episiotomy	0.74	0.69; 0.79	< 0.001
Instrumental vaginal birth	0.76	0.68; 0.86	< 0.001
Instrumental vaginal birth with fundal pressure	0.52	0.46; 0.59	< 0.001
Planned cesarean birth	0.8	0.74; 0.87	< 0.001
Unplanned cesarean birth, before the onset of labor	0.6	0.54; 0.67	< 0.001
Unplanned cesarean birth, after the onset of labor	0.52	0.48; 0.56	< 0.001
Age			
18–24	0.81	0.73; 0.91	< 0.001
25–30	0.96	0.91; 1.01	0.124
31–35	Ref	Ref	
36–39	1.05	0.98; 1.12	0.178
> 40	1.05	0.94; 1.16	0.41
Parity			
Primiparous	Ref	Ref	
Multiparous	1.27	1.21; 1.34	< 0.001
Woman gave birth in the country she herself was born in			
Yes	Ref	Ref	
No	1.01	0.93; 1.1	0.813
Level of education			
Junior high school or lower	0.97	0.88; 1.08	0.626
High school	0.94	0.89; 1	0.046
University degree	Ref	Ref	
Postgraduate/master/doctorate	0.93	0.88; 0.99	0.013
Country			
Other^b^	0.98	0.81; 1.18	0.809
Austria	1.05	0.83; 1.34	0.667
Bosnia‐Herzegovina	0.6	0.47; 0.78	< 0.001
Croatia	0.6	0.54; 0.66	< 0.001
France	1.15	1; 1.33	0.056
Germany	1	0.86; 1.15	0.949
Greece	0.68	0.59; 0.77	< 0.001
Latvia	0.51	0.47; 0.57	< 0.001
Lithuania	0.45	0.39; 0.52	< 0.001
Luxembourg	1.03	0.83; 1.29	0.769
Norway	1.51	1.39; 1.65	< 0.001
Poland	0.91	0.8; 1.03	0.131
Portugal	0.91	0.79; 1.05	0.188
Romania	1.08	0.93; 1.26	0.313
Serbia	0.45	0.37; 0.55	< 0.001
Slovenia	0.9	0.8; 1	0.052
Spain	0.43	0.34; 0.55	< 0.001
Sweden	1.38	1.28; 1.5	< 0.001
Switzerland	1.15	1; 1.33	0.046
Italy	Ref	Ref	

^a^ Medical interventions: vaginal birth with episiotomy, instrumental vaginal birth, instrumental vaginal birth with fundal pressure, planned cesarean birth, unplanned cesarean birth before or after the onset of labor.

^b^ Other countries include: Albania, Armenia, Belgium, Bulgaria, Cyprus, Czech Republic, Denmark, Estonia, Finland, Hungary, Iceland, Ireland, Israel, Macedonia, Malta, Montenegro, Netherlands, Republic of Moldova, Slovakia, Turkey, and Ukraine.

Concerning the different birth modes and medical interventions, compared with women who reported a vaginal birth, those who had an instrumental vaginal birth were less likely to have a positive birth experience (aOR: 0.76; CI: 0.68; 0.86). Medical interventions such as episiotomies in women with vaginal birth (aOR: 0.74; CI: 0.69; 0.79) and fundal pressure in women with instrumental vaginal birth (aOR: 0.52; CI: 0.46; 0.59) were associated with decreased odds of a positive perception of birth. All types of cesarean births were negatively associated with positive birth perception, from women with planned cesarean births (aOR: 0.80; CI: 0.74; 0.87) to those with unplanned cesarean births before (aOR: 0.60; CI: 0.54; 0.67) and after the onset of labor (aOR: 0.52; CI: 0.48; 0.56). Women younger than 25 years (aOR: 0.81; CI: 0.73; 0.91) showed significantly lower odds of a positive perception of birth compared to women aged 25 or older. No further age effect was observed.

No significant difference was found between women giving birth in their own country of birth and those who gave birth in a foreign country. Women from Norway (aOR: 1.51; CI: 1.39; 1.65), Sweden (aOR: 1.38; CI: 1.28; 1.5), and Switzerland (aOR: 1.55; CI: 1.; 1.33) had significantly higher odds of experiencing their birth positively compared with women from Italy, whereas women from Spain (aOR: 0.43; CI: 0.34; 0.55), Lithuania (aOR: 0.45; CI: 0.39; 0.52), Latvia (aOR: 0.51; CI: 0.47; 0.57), Serbia (aOR: 0.45; CI: 0.37; 0.55), Greece (aOR: 0.68; CI: 0.59; 0.77), Croatia (aOR: 0.60; CI: 0.54; 0.66), and Bosnia‐Herzegovina (aOR: 0.60; CI: 0.47; 0.78) were more likely to have less positive birth perceptions than women from Italy. No significant differences for positive perception of birth were observed for Austria, France, Germany, Luxembourg, Poland, Portugal, Romania, and Slovenia, compared with women from Italy.

### 3.4 Sensitivity Analysis

The sensitivity analysis presented similar results to the primary logistic regression model and supported the association between the health professionals' attention, assistance, and availability; the presence of a birth companion of choice; and the effect of mode of birth and medical interventions on perception (Supporting Information: File S3).

## 4 Discussion

Given the significant changes health systems underwent during the COVID‐19 pandemic, this study's findings are particularly relevant. With data from more than 48,000 women in 20 WHO European Region countries, we underscore the importance of ensuring QMNC standards even during extraordinary situations such as the pandemic, as a way of ensuring maternal health and well‐being. We found that 40% of the women in our sample did not have a birth companion of choice, and this limitation was associated with a lower likelihood of positive birth perception. Increased experiences of disrespect and abuse were more frequent among women who did not have a birth companion of choice. Our findings are in line with existing research, indicating that the presence of a birth companion of choice can be a protective factor against disrespect and abuse during childbirth [25]. Our findings also align with previous research highlighting the manyfold benefits of birth companions [40].

Our study also aligns with research showing that respectful maternity care is crucial and linked to positive birth experiences [41, 42]. Research shows that staff shortages and COVID‐19 containment measures negatively impacted the delivery of respectful maternity care, as reported by health professionals [43] and women [44]. In anticipation of future crises, it is imperative to develop guidelines that assess the best‐available evidence to protect the health and well‐being of both healthcare providers and birthing women, while maintaining quality, respectful maternity care, including ensuring a birth companion of choice whenever possible.

Our study indicates that women who consistently receive attention, assistance, and have access to an adequate number of health professionals are most likely to have a positive perception of their birth. The importance of a labor companion and attentive care is vital in crises and during normal times, improving maternal care quality and postpartum health outcomes by fostering positive birth experiences and preventing traumatic birth experiences [45, 46, 47].

Our research also adds to the existing literature [48, 49] by showing an association between the mode of birth and women's perceptions. Women with vaginal births tended to report more positive birth perceptions, whereas births involving higher degrees of medical intervention, such as instrumental vaginal birth, the use of fundal pressure, or unplanned cesarean birth initiated after the onset of labor, were often associated with less positive perceptions of birth. This is likely a reflection of the potentially stress‐inducing conditions surrounding (unplanned) interventions and a birth mode not aligned with the birthing woman's expectations.

### 4.1 Strengths and Limitations of This Study

The development, validation, implementation, and translation of the survey, as well as the data collection and analysis processes, have been thoroughly detailed in previous publications by the IMAgiNE study group [28, 30]. A notable strength of this study is the sample size, which provides a comprehensive dataset of birthing experiences from women across Europe.

Our online study may have a selection bias, attracting participants with higher levels of education, better internet access, and a willingness to share their birth experiences, both positive and negative [50]. Methodological choices might have led to underreporting of medical interventions (e.g., fundal pressure and episiotomies were queried only in specific birth contexts). The questionnaire has recently been revised for clearer differentiation of interventions and birth modes to overcome this issue. Another limitation is related to the analytical framework of this study. The composite outcome for positive birth perception was not directly measured with specifically designed questions. Instead, it was deduced from a combination of variables selected by the authors, based on relevant literature, which are believed to adequately represent aspects of a positive birth perception.

### 4.2 Conclusion

Our study offers important new evidence by presenting associations between the positive perception of birth, the level of professional support provided to women, and the presence of a chosen companion during labor and birth in 20 countries. It suggests that it is crucial to ensure an adequate number of health professionals available to deliver high‐quality and respectful maternity care and ensure that women have a companion of choice throughout labor and birth. This new evidence will hopefully contribute to increased investments in maternity care that place a high value on the perception that women have of their labor and birth.

## 5 Disclaimer

The authors alone are responsible for the views expressed in this article, and they do not necessarily represent the views, decisions, or policies of the institutions with which they are affiliated.

## Conflicts of Interest

The authors declare no conflicts of interest.

## Supporting information


File S1.

File S2.

File S3.

## Data Availability

The data that support the findings of this study are available on request from the corresponding author. The data are not publicly available due to privacy or ethical restrictions.

## Appendix A IMAgiNE EURO Study Group

**Austria:** Martina König‐Bachmann^1^, Christoph Zenzmaier^1^, Imola Simon^2^, Elisabeth D'Costa^3^ – ^1^Health University of Applied Sciences, Innsbruck, Austria, ^2^University of Applied Sciences Burgenland, Pinkafeld, Austria, ^3^Medical University of Innsbruck, Innsbruck, Austria. **Belgium:** Anne Galle^1^, Silke DHauwers^1^ – ^1^UCVV, University Center Nursery and Midwifery at Ghent University. **Bosnia‐Herzegovina:** Amira Ćerimagić^1^ – ^1^NGO Baby Steps, Sarajevo, Bosnia‐Herzegovina. **Cyprus:** Ourania Kolokotroni^1^, Eleni Hadjigeorgiou^1^, Maria Karanikola^1^, Nicos Middleton^1^, Ioli Orphanide Eteocleous^2^ – ^1^Cyprus University of Technology School of Health Sciences, ^2^Birth Forward NGO. **Croatia:** Daniela Drandić^1^, Magdalena Kurbanović^2^ – ^1^Roda – Parents in Action, Zagreb, Croatia, ^2^Faculty of Health Studies, University of Rijeka, Rijeka, Croatia. **Czech Republic:** Lenka Laubrova Zirovnicka^1^, Miloslava Kramná^2^ – ^1^Association For Freestanding Birth Centres and Alongside Midwifery Units (APODAC), ^2^Healthy Parenting Association (APERIO). **France:** Rozée Virginie^1^, Elise de La Rochebrochard^1^, Kristina Löfgren^2^ – ^1^Sexual and Reproductive Health and Rights Research Unit, Institut National d’Études Démographiques (INED), Aubervilliers, France, ^2^Baby‐friendly Hospital Initiative (IHAB), France. **Germany:** Céline Miani^1^, Stephanie Batram‐Zantvoort^1^ – ^1^Department of Epidemiology and International Public Health, School of Public Health, Bielefeld University, Bielefeld, Germany. **Greece:** Antigoni Sarantaki^1^, Dimitra Metallinou^1^, Aikaterini Lykeridou^1^ – ^1^Department of Midwifery, School of Health and Care Sciences, University of West Attica, Athens, Greece. **Israel:** Ilana Chertok^1,2^, Rada Artzi‐Medvedik^1^ – ^1^Ohio University, School of Nursing, Athens, Ohio, USA, ^2^Ruppin Academic Center, Department of Nursing, Emek Hefer, Israel **Italy:** Marzia Lazzerini^1^, Emanuelle Pessa Valente^1^, Ilaria Mariani^1^, Arianna Bomben^1^, Stefano Delle Vedove^1^, Sandra Morano^3^, Antonella Nespoli^3^, Simona Fumagalli^3^ – ^1^Institute for Maternal and Child Health IRCCS Burlo Garofolo, Trieste, Italy, ^2^Medical School and Midwifery School, Genoa University, Genoa, Italy, ^3^University of Milano Bicocca, Italy. **Latvia**: Elizabete Pumpure^1,2^, Dace Rezeberga^1,2,3^, Dārta Jakovicka^4,5^, Gita Jansone‐Šantare ^1,2^, Anna Šibalova^1^, Elīna Voitehoviča ^1,3^, Dārta Krēsliņa^1^ – ^1^Department of Obstetrics and Gynecology, Rīga Stradiņš University, Rīga, Latvia, ^2^Riga East Clinical University Hospital, Rīga, Latvia, ^3^Riga Maternity Hospital, Rīga, Latvia, ^4^Rīga Stradiņš University, Rīga, Latvia, ^5^Children's Clinical University Hospital, Rīga, Latvia. **Lithuania:** Alina Liepinaitienė^1,2,3^, Andželika Kondrakova^2^, Marija Mizgaitienė^4^, Simona Juciūtė^5^ – ^1^Faculty of Natural Sciences, Department of Environmental Sciences, Vytautas Magnus University, Kaunas, Lithuania, ^2^Kauno kolegija Higher Education Institution, Kaunas, Lithuania, ^3^Republican Siauliai County Hospital, Siauliai, Lithuania, ^4^Kaunas Hospital of the Lithuanian University of Health Sciences, Kaunas, Lithuania, ^5^Hospital of Lithuanian University of Health Sciences Kauno klinikos, Kaunas, Lithuania. **Luxembourg:** Maryse Arendt^1^, Barbara Tasch^1,2^ – ^1^Beruffsverband vun de Laktatiounsberoderinnen zu Lëtzebuerg asbl (Professional association of the Lactation Consultants in Luxembourg), Luxembourg, Luxembourg, ^2^Neonatal intensive care unit, KannerKlinik, Centre Hospitalier de Luxembourg, Luxembourg, Luxembourg. **Netherlands:** Enrico Lopriore^1^, Thomas Van den Akker^1,2^ – ^1^Leiden University Medical Center, Leiden, the Netherlands, ^2^Athena Institute, Vrije Universiteit, Amsterdam, Netherlands. **Norway**: Ingvild Hersoug Nedberg^1^, Sigrun Kongslien^1^, Eline Skirnisdottir Vik^2^ – ^1^Department of health and care sciences, UiT The Arctic University of Norway, Norway, ^2^Department of health and caring sciences, Western Norway University of Applied Sciences, Norway **Poland:** Barbara Baranowska^1^, Urszula Tataj‐Puzyna^1^, Beata Szlendak^1^, Paulina Pawlicka^2^ – ^1^Department of Midwifery, Centre of Postgraduate Medical Education, Warsaw, Poland, ^2^Division of Intercultural Psychology and Gender Psychology, University of Gdańsk, Gdańsk, Poland **Portugal:** Raquel Costa^1,2,3^, Catarina Barata^4^, Teresa Santos^5,6^, Heloísa Dias^7^, Tiago Miguel Pinto^3^, Sofia Marques^8,9^, Ana Meireles^8,9^, Joana Oliveira^8,9^, Mariana Pereira^9^, Maria Arminda Nunes^10,11^ – ^1^EPIUnit ‐ Instituto de Saúde Pública, Universidade do Porto, Porto, Portugal, ^2^Laboratório para a Investigação Integrativa e Translacional em Saúde Populacional (ITR), Universidade do Porto, Porto, Portugal, ^3^Lusófona University, HEI‐Lab: Digital Human‐Environment Interaction Labs, Portugal, ^4^Instituto de Ciências Sociais, Universidade de Lisboa, Lisboa, Portugal and Associação Portuguesa pelos Direitos da Mulher na Gravidez e Parto, Portugal, ^5^Universidade Europeia, Lisboa, Portugal, ^6^Plataforma CatólicaMed/Centro de Investigação Interdisciplinar em Saúde (CIIS) da Universidade Católica Portuguesa, Lisbon, Portugal, ^7^Regional Health Administration of the Algarve, (ARS ‐ Algarve, IP), Portugal, ^8^Institute of Psychology and Educational Sciences, Lusíada University, Porto, Portugal, ^9^CIPD—Psychology for Development Research Centre, Lusíada University, Porto, Portugal, ^10^Associação Portuguesa dos Enfermeiros Obstetras, Portugal, ^11^Nursing School of Porto, Porto, Portugal. **Romania:** Marina Ruxandra Otelea^1,2^ – ^1^University of Medicine and Pharmacy Carol Davila, Bucharest, Romania, ^2^SAMAS Association, Bucharest, Romania **Serbia**: Jelena Radetić^1^, Jovana Ružičić^1^ – ^1^Centar za mame, Belgrade, Serbia. **Slovenia**: Zalka Drglin^1^, Anja Bohinec^1^ – ^1^National Institute of Public Health, Ljubljana, Slovenia. **Spain:** Serena Brigidi^1^, Alejandra Oliden^2^, Lara Martín Castañeda^1,2^ – ^1^Institute of Research (VHIR), Vall d'Hebron University Foundation. Maternal and Fetal Medicine Research Group Medical Anthropology Research Center ‐ MARC ‐ Rovira i Virgili University, Tarragona, Spain. Department of Anthropology, Philosophy, and Social Work ‐ Rovira i Virgili University, Tarragona, Spain. President of Observatory of Obstetric Violence in Spain ‐ OVO, ^2^Nurse, La casa de Isis Birth Centre ‐ Orba, Alicante, Spain Member of Observatory of Obstetric Violence in Spain – OVO. **Sweden**: Helen Elden^1,2^, Karolina Linden^1^, Mehreen Zaigham^3^ – ^1^Institute of Health and Care Sciences, Sahlgrenska Academy, University of Gothenburg, Gothenburg, Sweden, ^2^Department of Obstetrics and Gynecology, Region Västra Götaland, Sahlgrenska University Hospital, Gothenburg, Sweden, ^3^Obstetrics and Gynecology, Department of Obstetrics and Gynecology, Institution of Clinical Sciences Lund, Lund University, Lund and Skåne University Hospital, Malmö, Sweden. **Switzerland**: Claire de Labrusse^1^, Alessia Abderhalden‐Zellweger^1^, Anouck Pfund^1^, Harriet Thorn^1^, Susanne Grylka^2^, Michael Gemperle^2^, Antonia Mueller^2^ – ^1^School of Health Sciences (HESAV), HES‐SO University of Applied Sciences and Arts Western Switzerland, Lausanne, Switzerland, ^2^Institute of Midwifery and Reproductive Health, School of Health Sciences, ZHAW Zurich University of Applied Sciences. **TAG member**: Emma Sacks, Department of International Health, Johns Hopkins School of Public Health, Baltimore, Maryland, USA.

## References

[1] K. Coxon , C. F. Turienzo , L. Kweekel , et al., “The Impact of the Coronavirus (COVID‐19) Pandemic on Maternity Care in Europe,” Midwifery 88 (2020): 102779. 32600862 10.1016/j.midw.2020.102779 PMC7286236

[2] T. S. Eri , E. Blix , S. Downe , C. Vedeler , and A. B. V. Nilsen , “Giving Birth and Becoming a Parent During the COVID‐19 Pandemic: A Qualitative Analysis of 806 Women's Responses to Three Open‐Ended Questions in an Online Survey,” Midwifery 109 (2022): 103321. 35349790 10.1016/j.midw.2022.103321 PMC8935971

[3] World Health Organization , Companion of Choice During Labour and Childbirth for Improved Quality of Care (World Health Organization, 2020).

[4] White Ribbon Alliance , Respectful Maternity Care (Universal Rights of Childbearing women, 2011).

[5] D. Drandić , K. Hartmann , C. Barata , and R. Torguet , “Parent Organizations' Experiences of the Pandemic Response in Maternity Care in Thirteen European Countries,” European Journal of Midwifery 6, no. December (2022): 1–10. 10.18332/ejm/156902 PMC9773267 36591331

[6] K. S. Arora , J. T. Mauch , and K. S. Gibson , “Labor and Delivery Visitor Policies During the COVID‐19 Pandemic: Balancing Risks and Benefits,” Journal of the American Medical Association 323, no. 24 (2020): 2468–2469. 32442264 10.1001/jama.2020.7563 PMC7736929

[7] “Women's Rights in Childbirth Must be Upheld During the Coronavirus Pandemic [press release].” 29.03.2020 2020.

[8] “DGGG Empfiehlt: Begleitung bei der Geburt zulassen – Auch in Zeiten der Corona‐Pandemie [press release].” 26.03.2020 2020.

[9] “Position Statement Delle Società Scientifiche e Delle Federazioni Professionali Sanitarie: Presenza Del Partner/Caregiver Nelle Aree di Recovero di Madri e Neonati in Corso de Pandemia da COVID‐19 [press release].” 13.12.2021. 2021.

[10] “Covid‐19 ‐ Préconisations du CNSF Pour la Pratique des Sages‐Femmes en Période de Confinement [press release].” 01.11.2020 2020.

[11] S. Oddo‐Sommerfeld , K. Schermelleh‐Engel , M. Konopka , V. L. La Rosa , F. Louwen , and S. Sommerlad , “Giving Birth Alone Due to COVID‐19‐Related Hospital Restrictions Compared to Accompanied Birth: Psychological Distress in Women With Caesarean Section or Vaginal Birth – A Cross‐Sectional Study,” Journal of Perinatal Medicine 50, no. 5 (2022): 539–548. 35357796 10.1515/jpm-2021-0368

[12] A. Nespoli , S. Ornaghi , S. Borrelli , P. Vergani , and S. Fumagalli , “Lived Experiences of the Partners of COVID‐19 Positive Childbearing Women: A Qualitative Study,” Women and Birth 35, no. 3 (2022): 289–297. 34353766 10.1016/j.wombi.2021.07.006 PMC9051041

[13] Y. K. Mok , K. W. Cheung , W. Wang , R. H. W. Li , N. W. M. Shek , and E. H. Yu Ng , “The Effects of Not Having Continuous Companion Support During Labour on Pregnancy and Neonatal Outcomes During the COVID‐19 Pandemic,” Midwifery 108 (2022): 103293. 35240431 10.1016/j.midw.2022.103293 PMC8860747

[14] S. J. Flaherty , H. Delaney , K. Matvienko‐Sikar , and V. Smith , “Maternity Care During COVID‐19: A Qualitative Evidence Synthesis of Women's and Maternity Care Providers' Views and Experiences,” BMC Pregnancy and Childbirth 22, no. 1 (2022): 438. 35619069 10.1186/s12884-022-04724-w PMC9132752

[15] M. A. Bohren , B. O. Berger , H. Munthe‐Kaas , and O. Tuncalp , “Perceptions and Experiences of Labour Companionship: A Qualitative Evidence Synthesis,” Cochrane Database of Systematic Reviews 3, no. 3 (2019): CD012449. 30883666 10.1002/14651858.CD012449.pub2 PMC6422112

[16] World Health Organization , “WHO Recommendations: Intrapartum Care for a Positive Childbirth Experience.” 2018. 30070803

[17] Wordl Health Organization , “WHO Recommendations on Health Promotion Interventions for Maternal and Newborn Health.” 2015. Report No.: 9789241508742. 26180864

[18] G. A. Sosa , K. E. Crozier , and A. Stockl , “Midwifery One‐To‐One Support in Labour: More Than a Ratio,” Midwifery 62 (2018): 230–239. 29727828 10.1016/j.midw.2018.04.016

[19] L. Page , “One‐To‐One Midwifery: Restoring the “With Woman” Relationship in Midwifery,” Journal of Midwifery & Women's Health 48, no. 2 (2003): 119–125. 10.1016/s1526-9523(02)00425-7 12686944

[20] L. Page , C. McCourt , S. Beake , A. Vail , and J. Hewison , “Clinical Interventions and Outcomes of One‐To‐One Midwifery Practice,” Journal of Public Health Medicine 21, no. 3 (1999): 243–248. 10528949 10.1093/pubmed/21.3.243

[21] M. A. Bohren , G. J. Hofmeyr , C. Sakala , R. K. Fukuzawa , and A. Cuthbert , “Continuous Support for Women During Childbirth,” Cochrane Database of Systematic Reviews 7, no. 4 (2017): CD003766. 28681500 10.1002/14651858.CD003766.pub6 PMC6483123

[22] L. Turner , D. Culliford , J. Ball , E. Kitson‐Reynolds , and P. Griffiths , “The Association Between Midwifery Staffing Levels and the Experiences of Mothers on Postnatal Wards: Cross Sectional Analysis of Routine Data,” Women and Birth 35, no. 6 (2022): e583‐e9. 35183474 10.1016/j.wombi.2022.02.005

[23] N. Schmitt , E. Mattern , E. Cignacco , et al., “Effects of the Covid‐19 Pandemic on Maternity Staff in 2020 ‐ a Scoping Review,” BMC Health Services Research 21, no. 1 (2021): 1364. 34961510 10.1186/s12913-021-07377-1 PMC8710925

[24] M. A. Bohren , J. P. Vogel , E. C. Hunter , et al., “The Mistreatment of Women During Childbirth in Health Facilities Globally: A Mixed‐Methods Systematic Review,” MIDIRS Midwifery Digest 26, no. 1 (2016): 129. 10.1371/journal.pmed.1001847 PMC4488322 26126110

[25] M. D. Balde , K. Nasiri , H. Mehrtash , et al., “Labour Companionship and Women's Experiences of Mistreatment During Childbirth: Results From a Multi‐Country Community‐Based Survey,” BMJ Global Health 5, no. Suppl 2 (2020): e003564, 10.1136/bmjgh-2020-003564. PMC7684665 33234502

[26] S. Batram‐Zantvoort , L. Wandschneider , O. Razum , and C. Miani , “A Critical Review: Developing a Birth Integrity Framework for Epidemiological Studies Through Meta‐Ethnography,” BMC Women's Health 23, no. 1 (2023): 530. 37817176 10.1186/s12905-023-02670-z PMC10565979

[27] S. Batram‐Zantvoort , O. Razum , and C. Miani , “Birth Integrity Through the Lens of Medicalization, Risk, Embodiment, and Intersectionality,” Santé Publique 33, no. 5 (2022): 645–654. 35724098 10.3917/spub.215.0645

[28] M. Lazzerini , B. Covi , I. Mariani , et al., “Quality of Facility‐Based Maternal and Newborn Care Around the Time of Childbirth During the COVID‐19 Pandemic: Online Survey Investigating Maternal Perspectives in 12 Countries of the WHO European Region,” Lancet Regulation Health Europe 13 (2022): 100268. 10.1016/j.lanepe.2021.100268 PMC8703114 34977838

[29] World Health Organization , Standards for Improving Quality of Maternal and Newborn Care in Health Facilities (World Health Organization, 2016).

[30] M. Lazzerini , G. Argentini , I. Mariani , et al., “WHO Standards‐Based Tool to Measure Women's Views on the Quality of Care Around the Time of Childbirth at Facility Level in the WHO European Region: Development and Validation in Italy,” BMJ Open 12, no. 2 (2022): e048195. 10.1136/bmjopen-2020-048195 PMC8852667 35172991

[31] M. Lazzerini , E. P. Valente , B. Covi , et al., “Rates of Instrumental Vaginal Birth and Cesarean and Quality of Maternal and Newborn Health Care in Private Versus Public Facilities: Results of the IMAgiNE EURO Study in 16 Countries,” International Journal of Gynaecology and Obstetrics 159, no. Suppl 1 (2022): 22–38. 36530007 10.1002/ijgo.14458 PMC10108180

[32] C. Miani , L. Wandschneider , S. Batram‐Zantvoort , et al., “Individual and Country‐Level Variables Associated With the Medicalization of Birth: Multilevel Analyses of IMAgiNE EURO Data From 15 Countries in the WHO European Region,” International Journal of Gynaecology and Obstetrics 159, no. Suppl 1 (2022): 9–21. 36530006 10.1002/ijgo.14459

[33] D. Drandic , Z. Drglin , B. Mihevc Ponikvar , et al., “Women's Perspectives on the Quality of Hospital Maternal and Newborn Care Around the Time of Childbirth During the COVID‐19 Pandemic: Results From the IMAgiNE EURO Study in Slovenia, Croatia, Serbia, and Bosnia‐Herzegovina,” International Journal of Gynaecology and Obstetrics 159, no. Suppl 1 (2022): 54–69. 36530003 10.1002/ijgo.14457 PMC9877897

[34] E. Pumpure , D. Jakovicka , I. Mariani , et al., “Women's Perspectives on the Quality of Maternal and Newborn Care in Childbirth During the COVID‐19 Pandemic in Latvia: Results From the IMAgiNE EURO Study on 40 WHO Standards‐Based Quality Measures,” International Journal of Gynaecology and Obstetrics 159, no. Suppl 1 (2022): 97–112. 10.1002/ijgo.14461 PMC9878132 36530013

[35] E. von Elm , D. G. Altman , M. Egger , et al., “The Strengthening the Reporting of Observational Studies in Epidemiology (STROBE) Statement: Guidelines for Reporting Observational Studies,” Journal of Clinical Epidemiology 61, no. 4 (2008): 344–349. 18313558 10.1016/j.jclinepi.2007.11.008

[36] C. Miani , A. Leisse , L. Wandschneider , and S. Batram‐Zantvoort , “Experiences of Giving Birth During the COVID‐19 Pandemic: A Qualitative Analysis of Social Media Comments Through the Lens of Birth Integrity,” BMC Pregnancy and Childbirth 23, no. 1 (2023): 32. 36647019 10.1186/s12884-022-05326-2 PMC9841489

[37] P. Carquillat , M. Boulvain , and M. J. Guittier , “How Does Delivery Method Influence Factors That Contribute to Women's Childbirth Experiences?,” Midwifery 43 (2016): 21–28. 27825057 10.1016/j.midw.2016.10.002

[38] F. Weeks , L. Pantoja , J. Ortiz , J. Foster , G. Cavada , and L. Binfa , “Labor and Birth Care Satisfaction Associated With Medical Interventions and Accompaniment During Labor Among Chilean Women,” Journal of Midwifery & Women's Health 62, no. 2 (2017): 196–203. 10.1111/jmwh.12499 27543442

[39] S. Fumagalli , E. Colciago , L. Antolini , A. Riva , A. Nespoli , and A. Locatelli , “Variables Related to Maternal Satisfaction With Intrapartum Care in Northern Italy,” Women and Birth 34, no. 2 (2021): 154–161. 32111557 10.1016/j.wombi.2020.01.012

[40] E. D. Hodnett , S. Gates , G. J. Hofmeyr , and C. Sakala , “Continuous Support for Women During Childbirth,” Cochrane Database of Systematic Reviews 10 (2012): CD003766. 12917986 10.1002/14651858.CD003766

[41] A. Miyauchi , E. Shishido , and S. Horiuchi , “Women's Experiences and Perceptions of Women‐Centered Care and Respectful Care During Facility‐Based Childbirth: A Meta‐Synthesis,” Japan Journal of Nursing Science 19, no. 3 (2022): e12475. 35133066 10.1111/jjns.12475

[42] E. Shakibazadeh , M. Namadian , M. A. Bohren , et al., “Respectful Care During Childbirth in Health Facilities Globally: A Qualitative Evidence Synthesis,” BJOG: An International Journal of Obstetrics and Gynaecology 125, no. 8 (2018): 932–942. 29117644 10.1111/1471-0528.15015 PMC6033006

[43] I. H. M. Jones , A. Thompson , C. L. Dunlop , and A. Wilson , “Midwives' and Maternity Support Workers' Perceptions of the Impact of the First Year of the COVID‐19 Pandemic on Respectful Maternity Care in a Diverse Region of the UK: A Qualitative Study,” BMJ Open 12, no. 9 (2022): e064731. 10.1136/bmjopen-2022-064731 PMC9490297 36127079

[44] M. Zaigham , K. Linden , V. Sengpiel , et al., “Large Gaps in the Quality of Healthcare Experienced by Swedish Mothers During the COVID‐19 Pandemic: A Cross‐Sectional Study Based on WHO Standards,” Women and Birth 35, no. 6 (2022): 619–627. 35123922 10.1016/j.wombi.2022.01.007 PMC8784577

[45] S. Nakic Rados , L. Martinic , M. Matijas , M. Brekalo , and C. R. Martin , “The Relationship Between Birth Satisfaction, Posttraumatic Stress Disorder and Postnatal Depression Symptoms in Croatian Women,” Stress and Health 38, no. 3 (2022): 500–508. 34762758 10.1002/smi.3112

[46] U. Nagle , S. Naughton , S. Ayers , S. Cooley , R. M. Duffy , and P. Dikmen‐Yildiz , “A Survey of Perceived Traumatic Birth Experiences in an Irish Maternity Sample ‐ Prevalence, Risk Factors and Follow Up,” Midwifery 113 (2022): 103419. 35930929 10.1016/j.midw.2022.103419

[47] S. Ayers , R. Bond , S. Bertullies , and K. Wijma , “The Aetiology of Post‐Traumatic Stress Following Childbirth: A Meta‐Analysis and Theoretical Framework,” Psychological Medicine 46, no. 6 (2016): 1121–1134. 26878223 10.1017/S0033291715002706

[48] P. Kempe and M. Vikstrom‐Bolin , “Women's Satisfaction With the Birthing Experience in Relation to Duration of Labour, Obstetric Interventions and Mode of Birth,” European Journal of Obstetrics, Gynecology, and Reproductive Biology 246 (2020): 156–159. 32028143 10.1016/j.ejogrb.2020.01.041

[49] S. Spaich , G. Welzel , S. Berlit , et al., “Mode of Delivery and Its Influence on Women's Satisfaction With Childbirth,” European Journal of Obstetrics, Gynecology, and Reproductive Biology 170, no. 2 (2013): 401–406. 23962715 10.1016/j.ejogrb.2013.07.040

[50] J. Bethlehem , “Selection Bias in Web Surveys,” International Statistical Review 78, no. 2 (2010): 161–188.

