# Supplementary figures and images for: The Association Between Women's Perception of Birth During the Pandemic, Companion of Choice and Support From Health Professionals: A Cross‐Sectional Study in 20 Countries in the WHO European Region
Source: Birth. 2025 Apr 4;52(4):677–89. doi: 10.1111/birt.12915 (PMC12612363; doi:10.1111/birt.12915)

**Supplementary file 2: Score distribution by country, and overall**

**
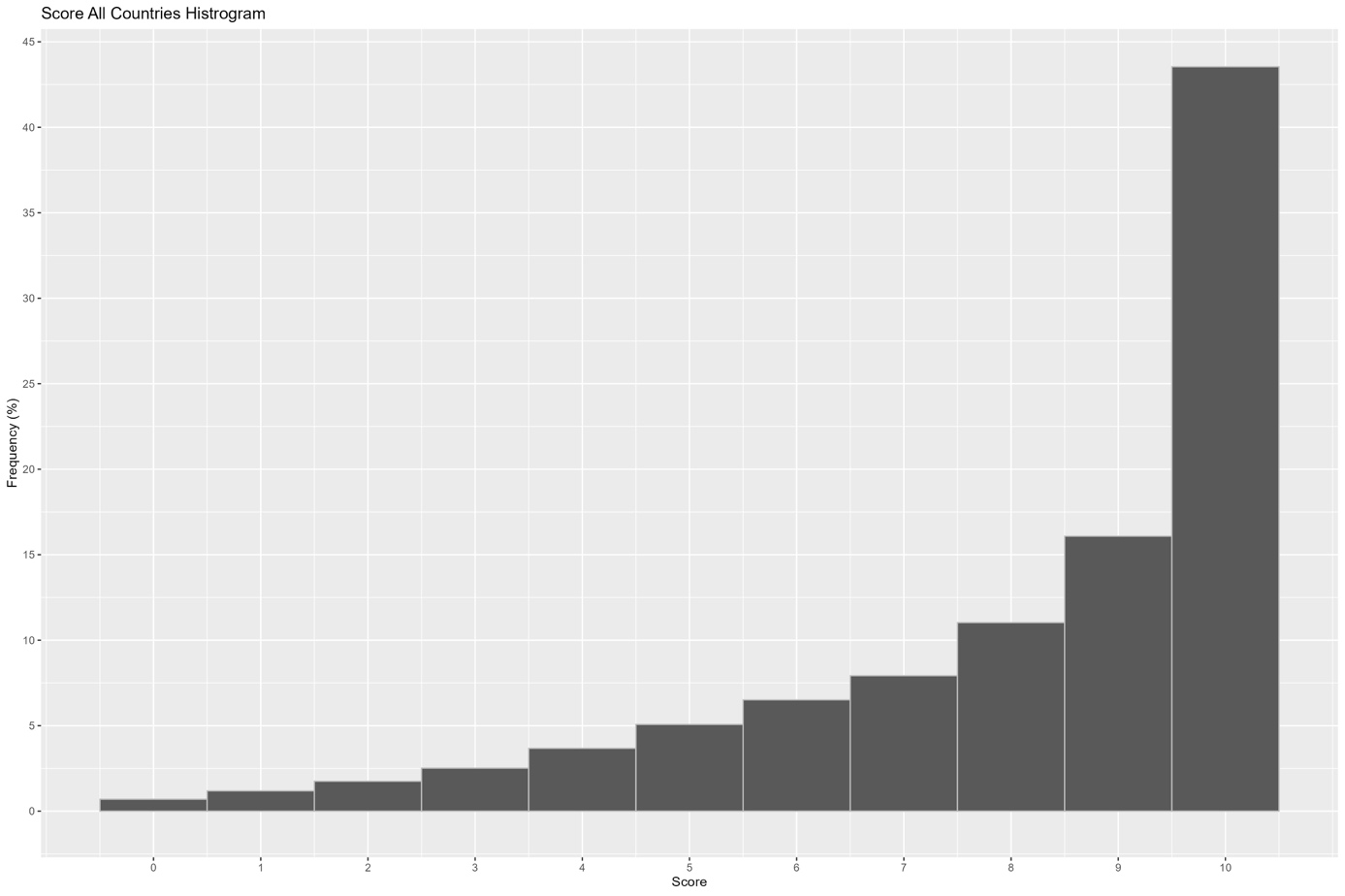
**

**
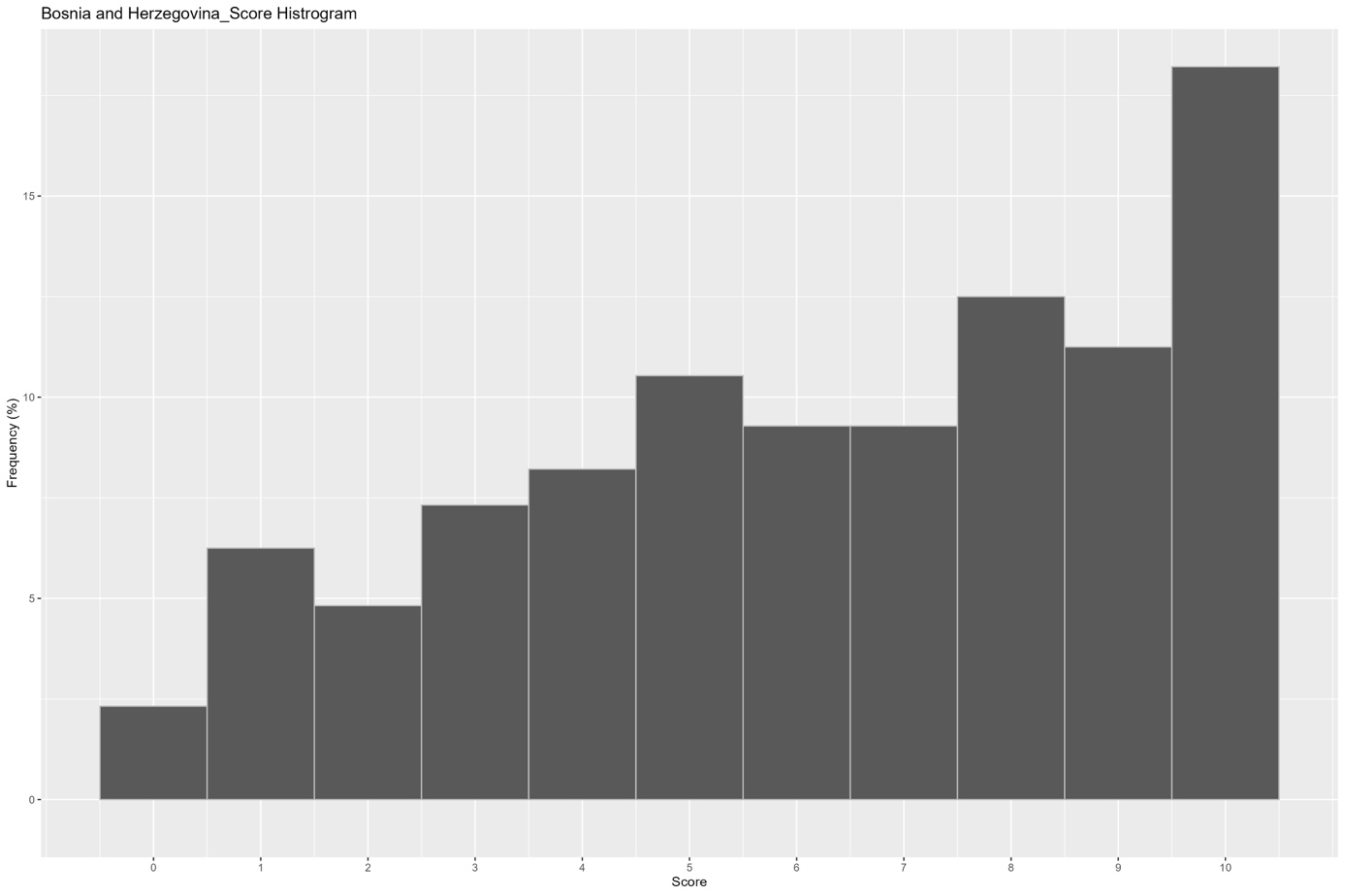

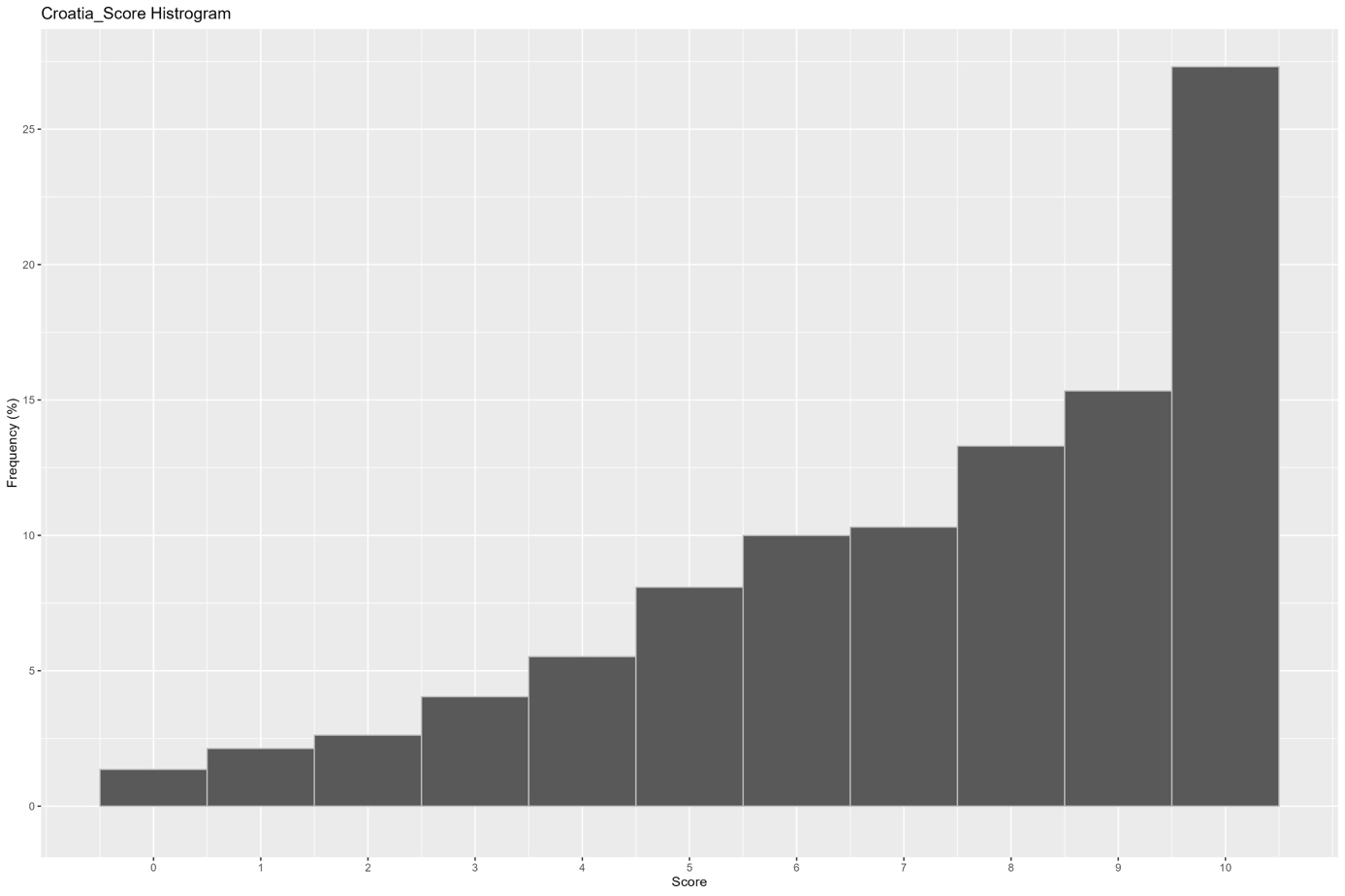

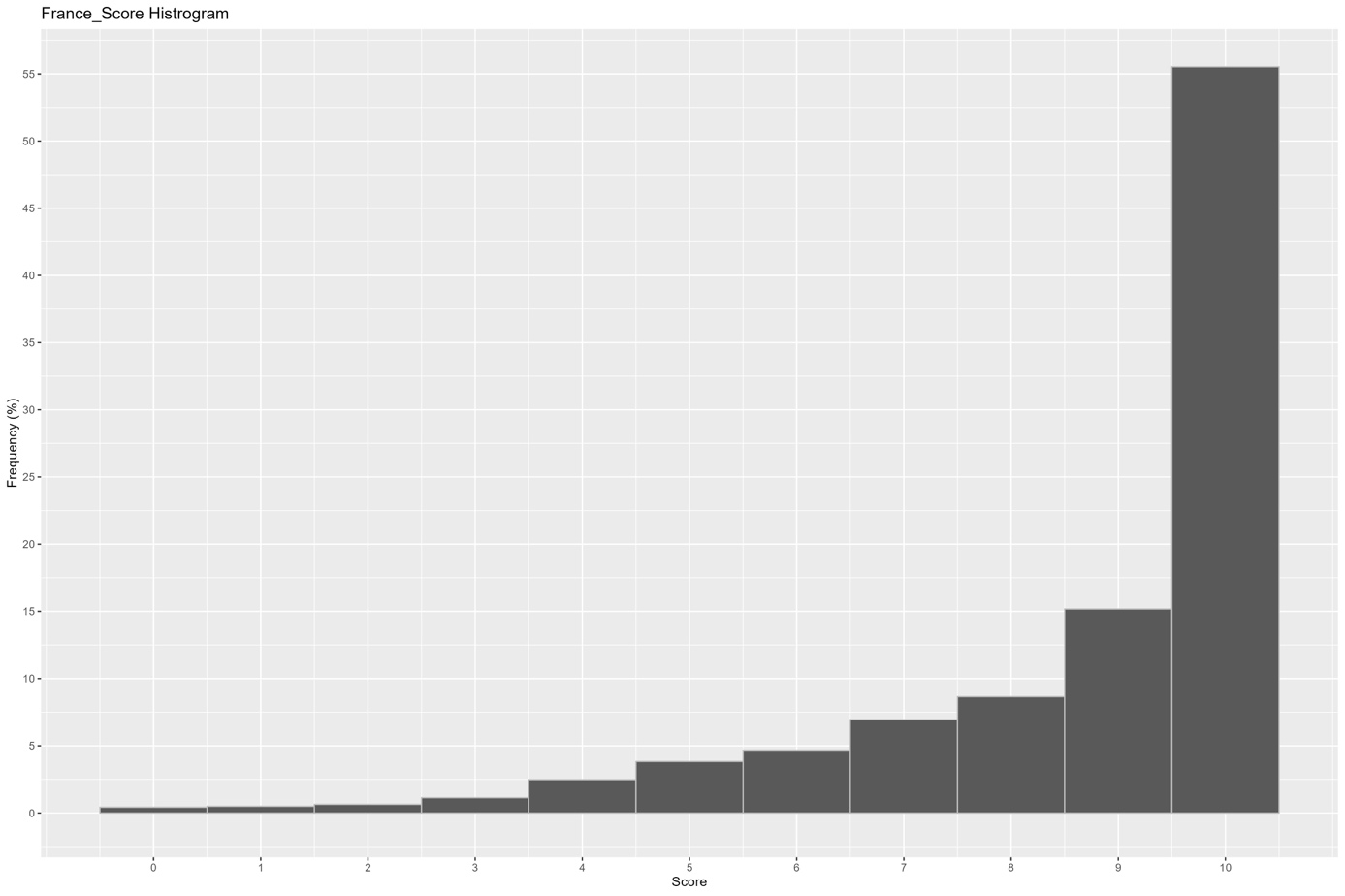

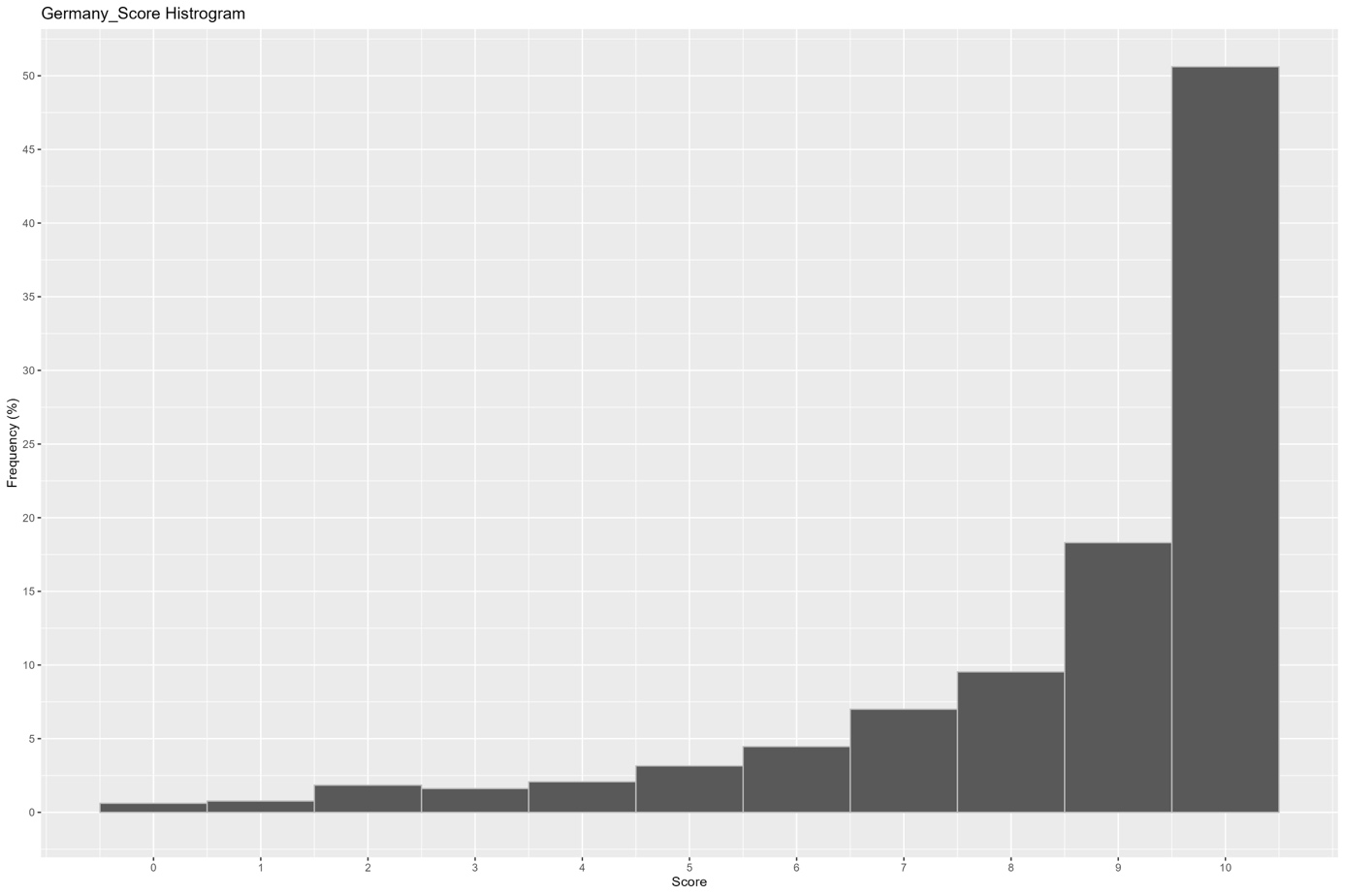

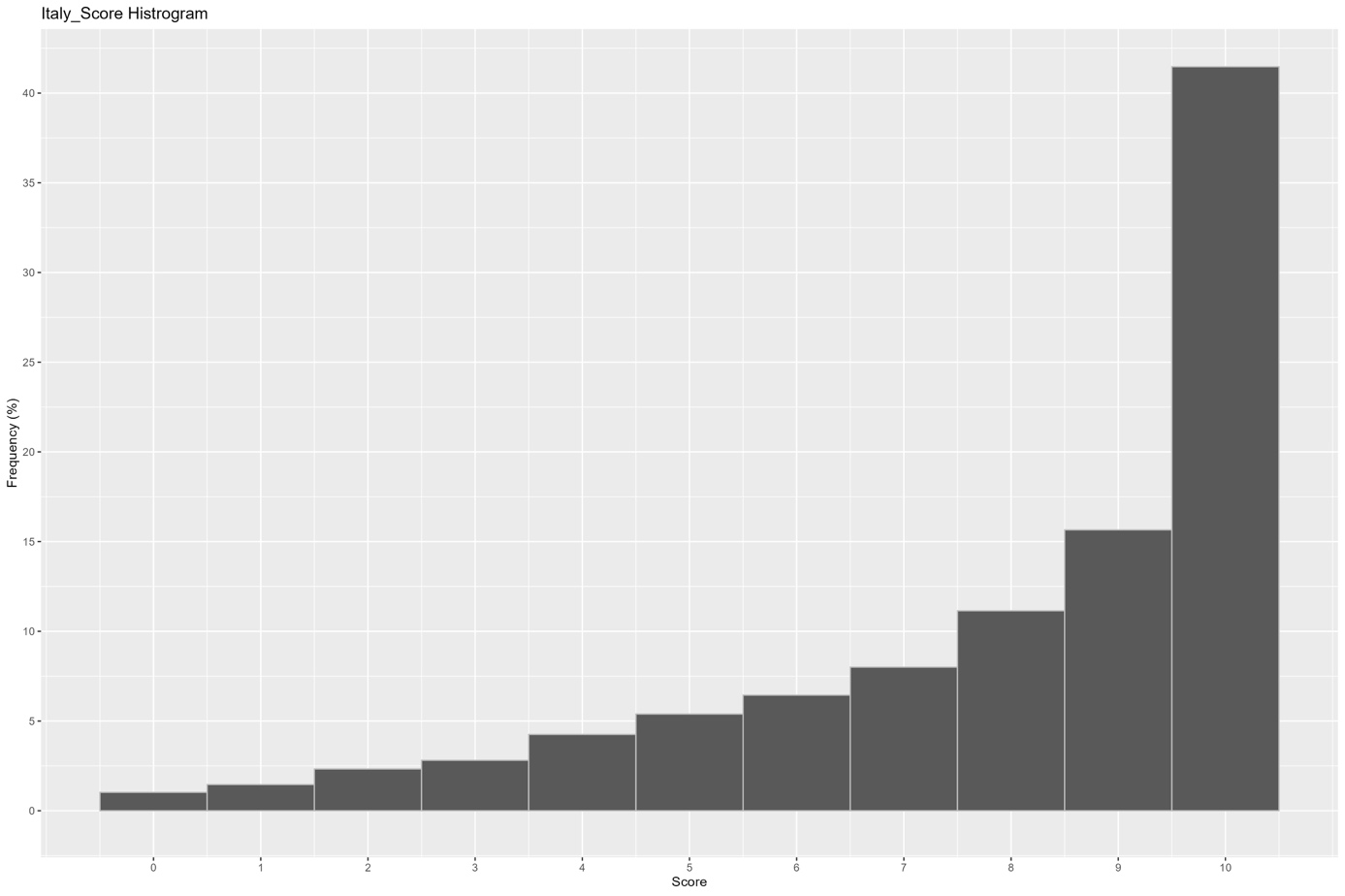

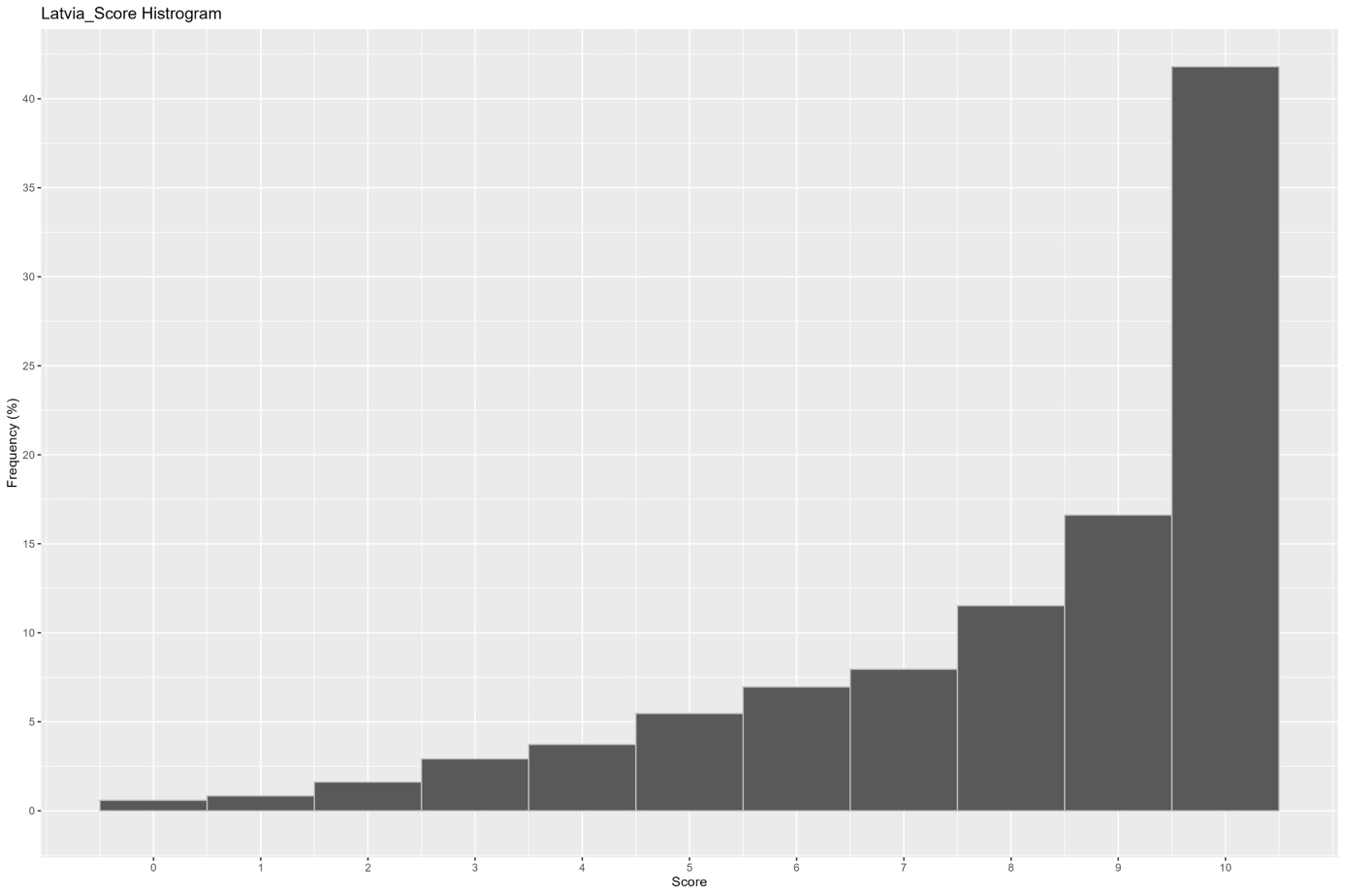

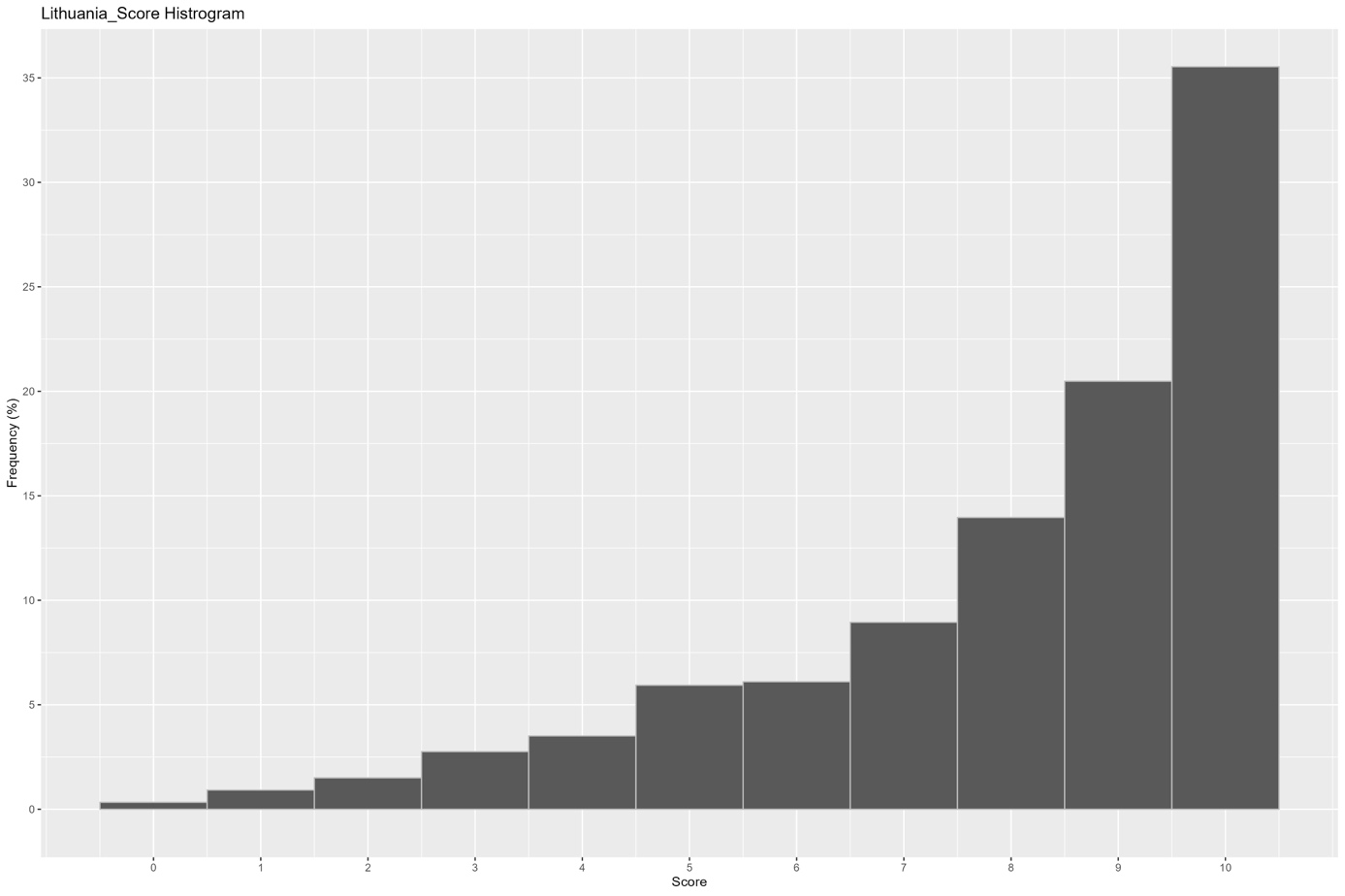

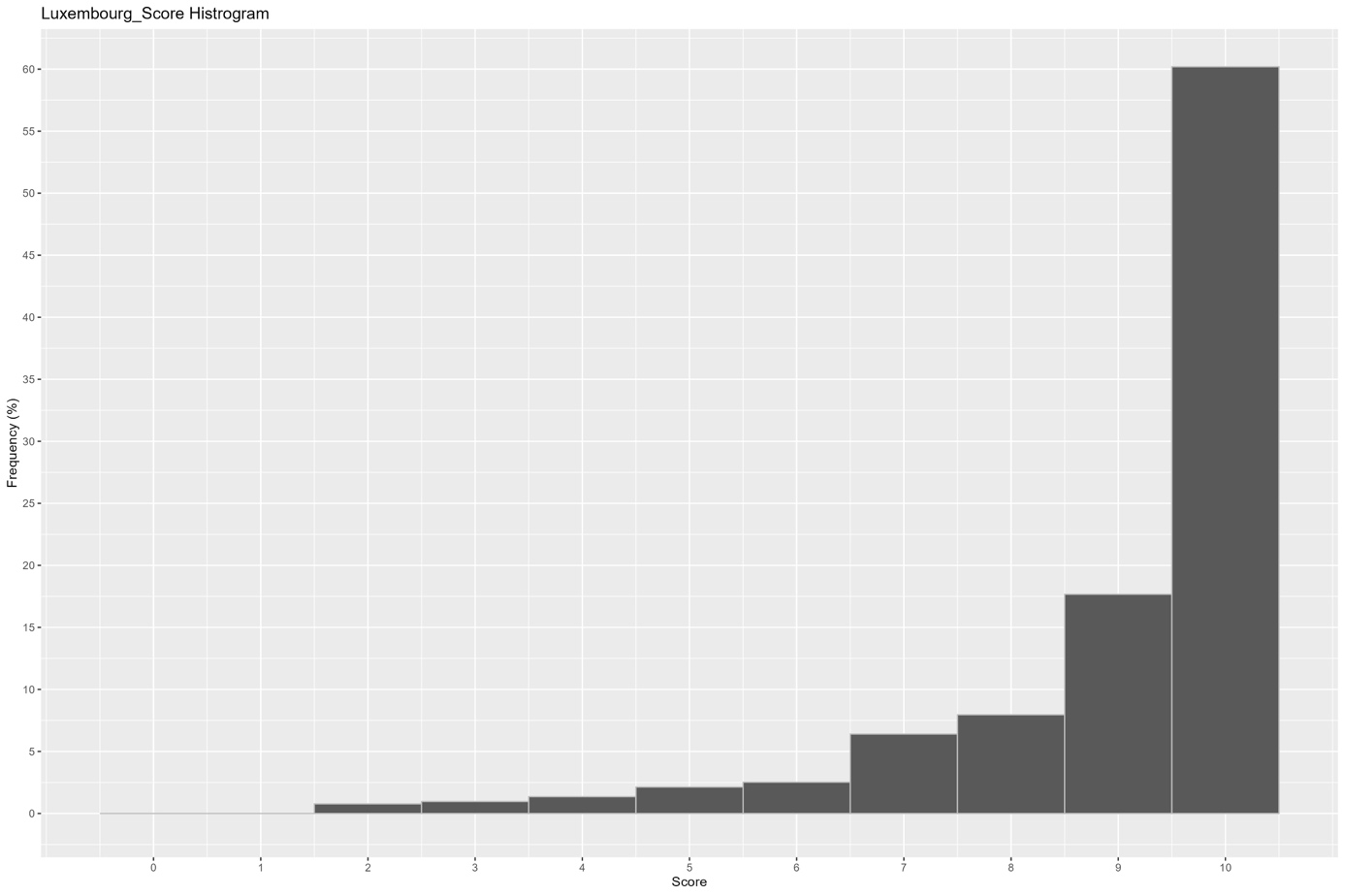

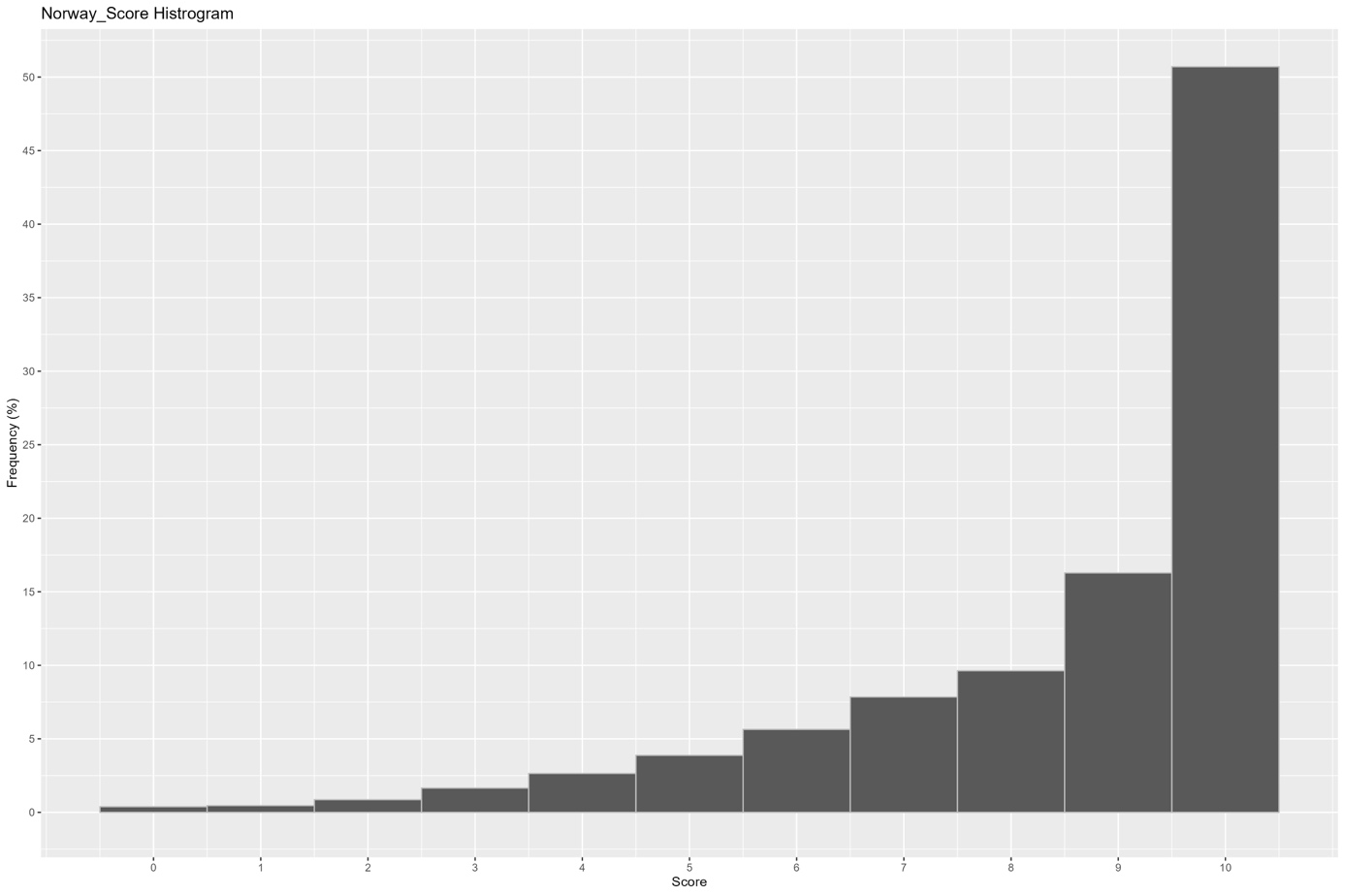

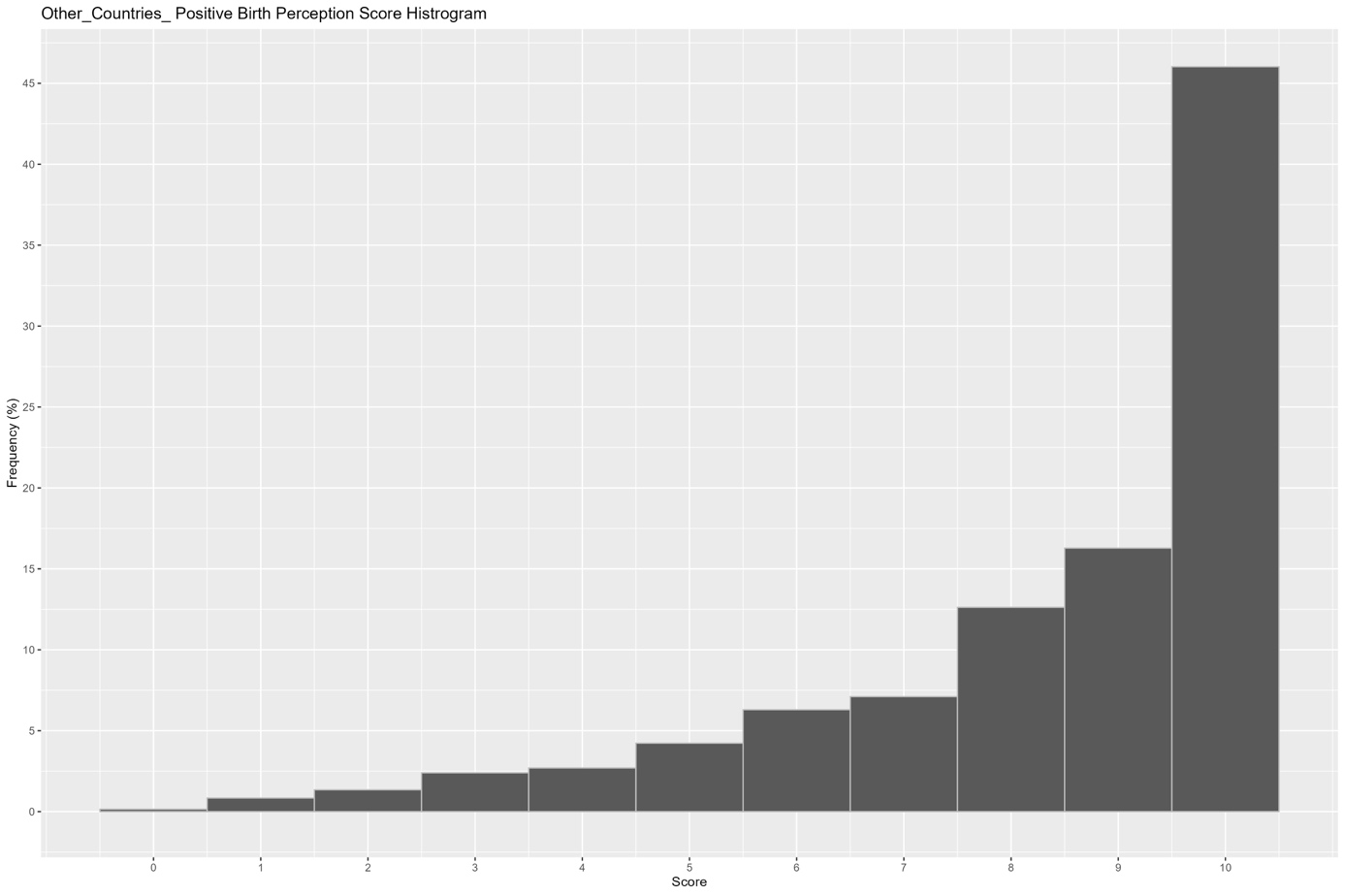

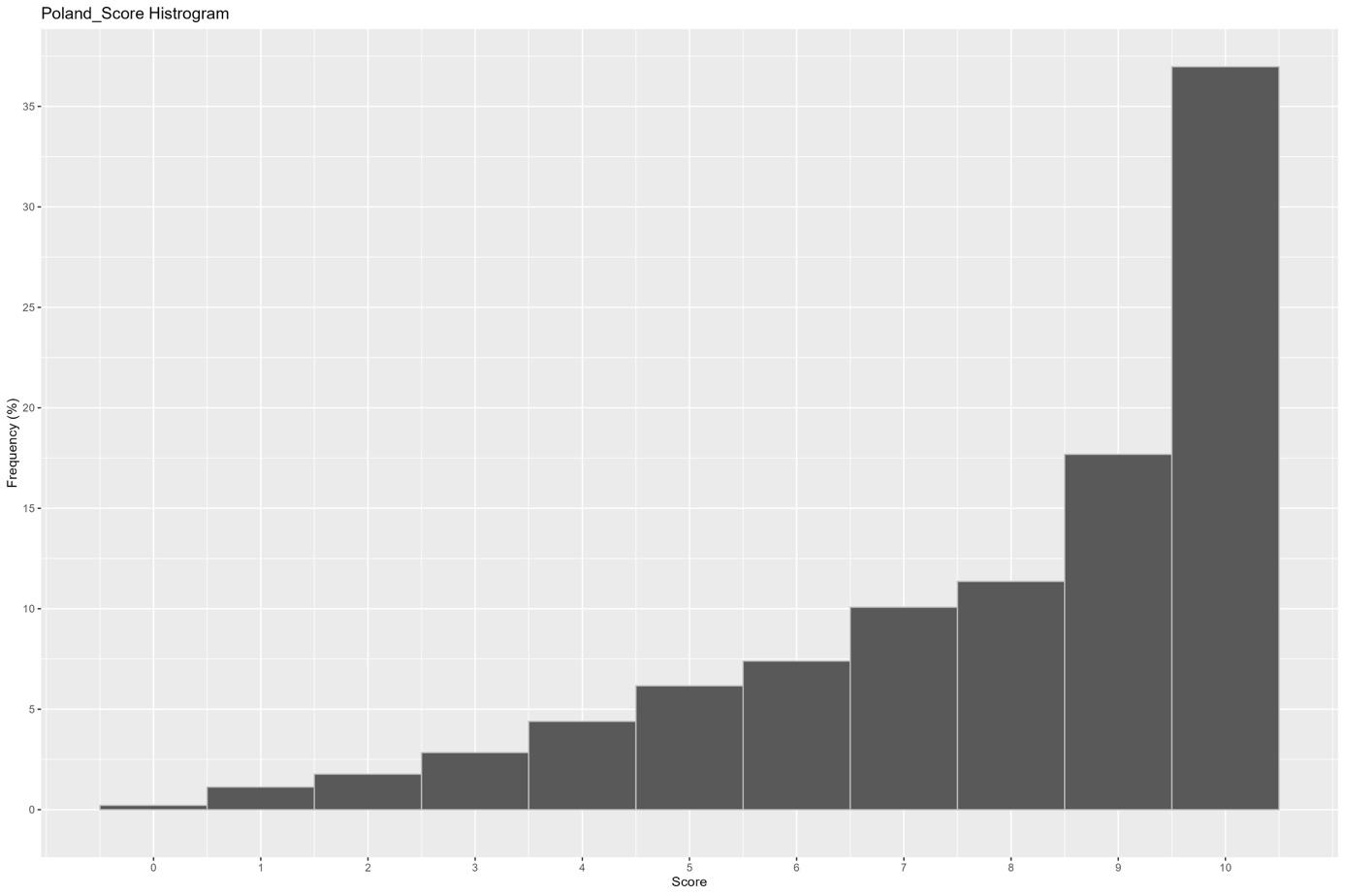

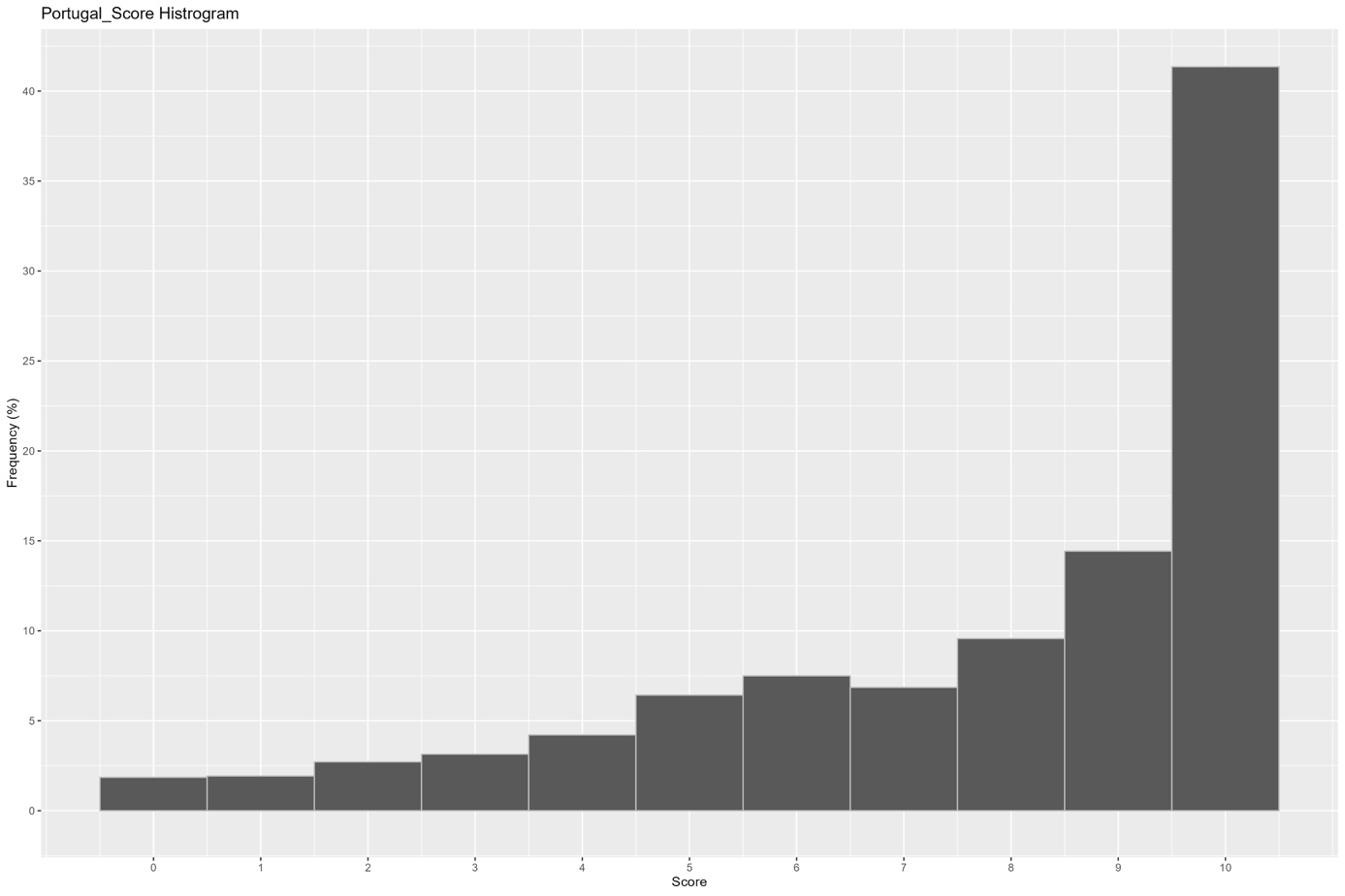

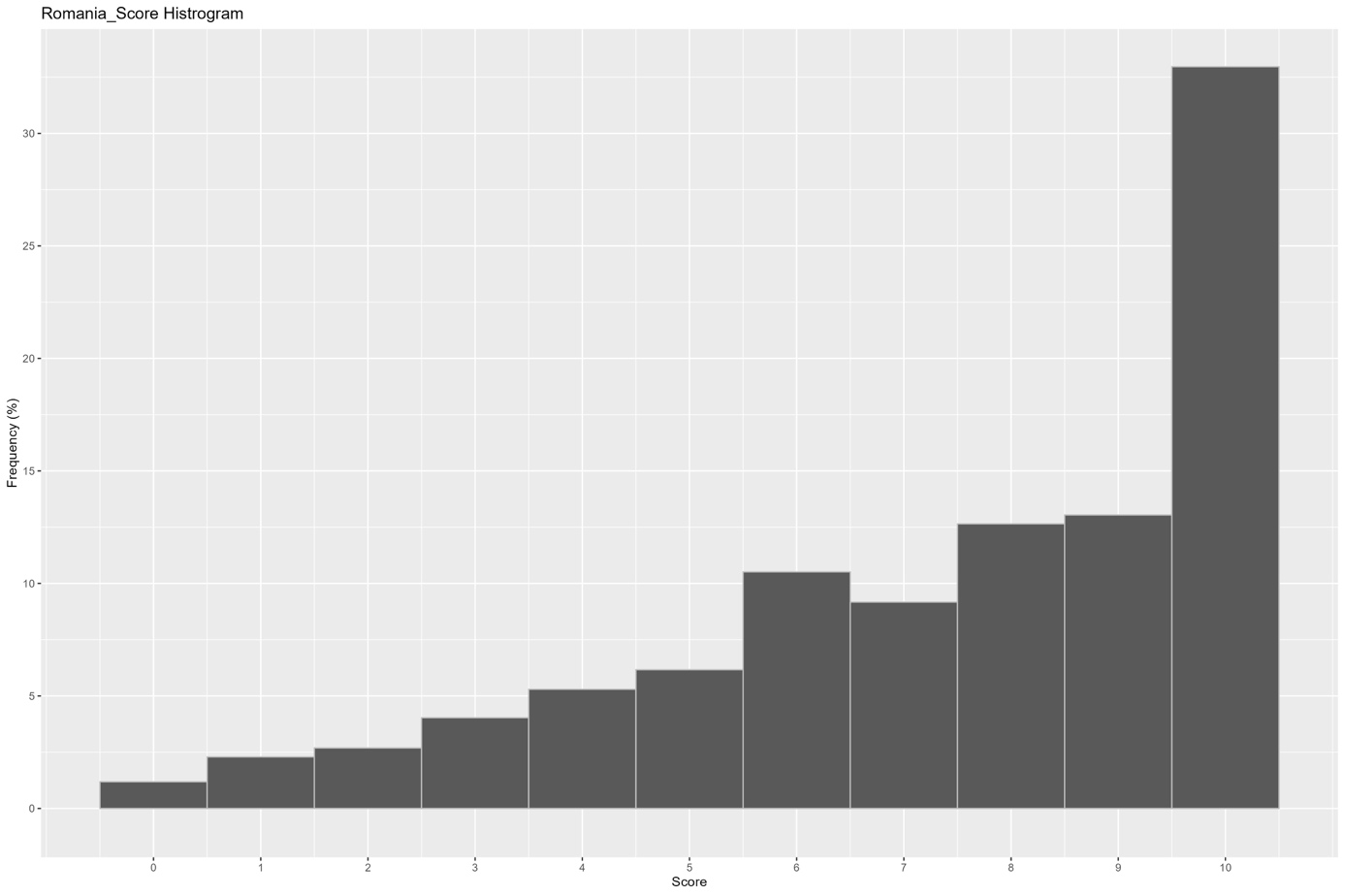

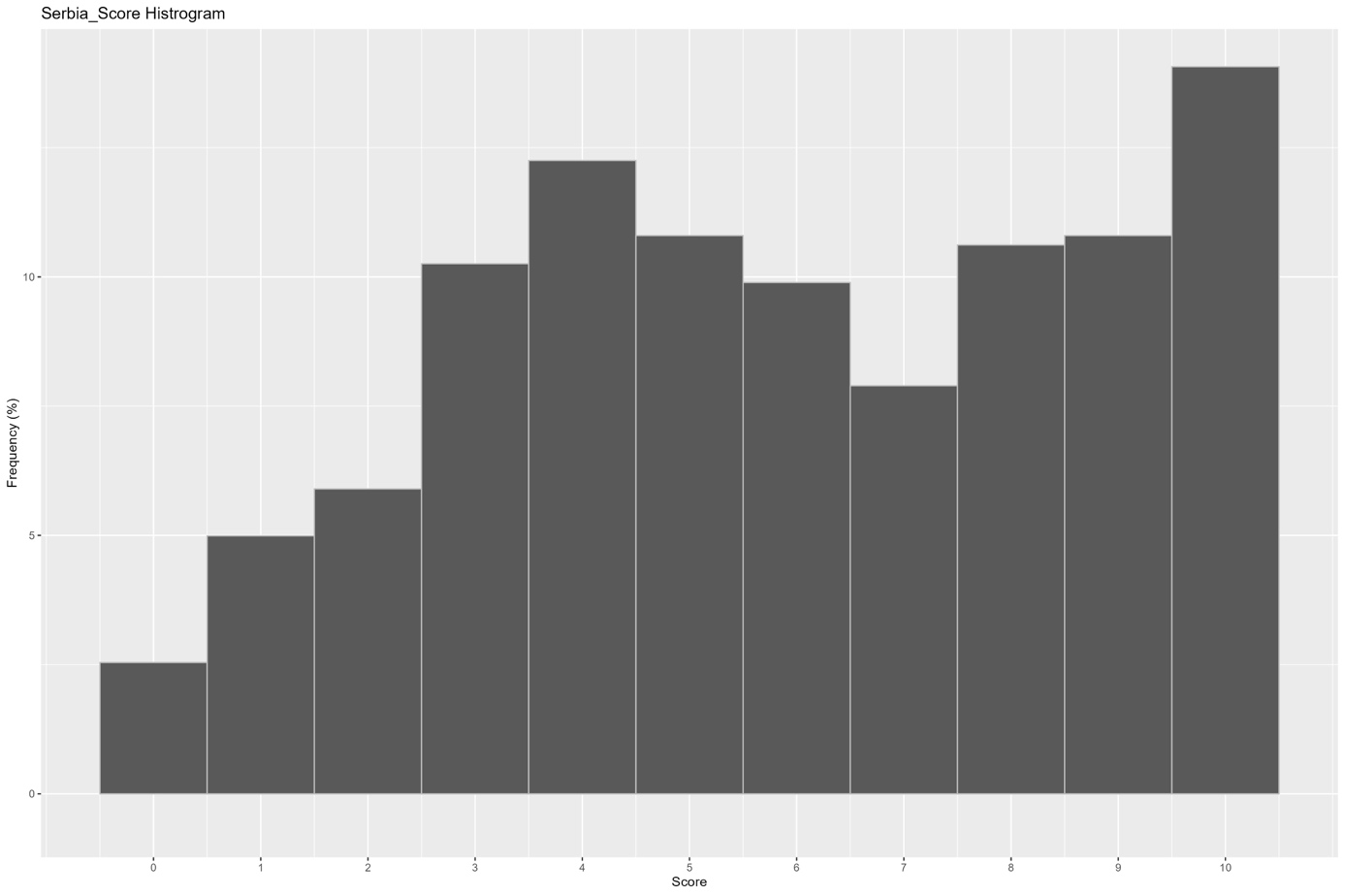

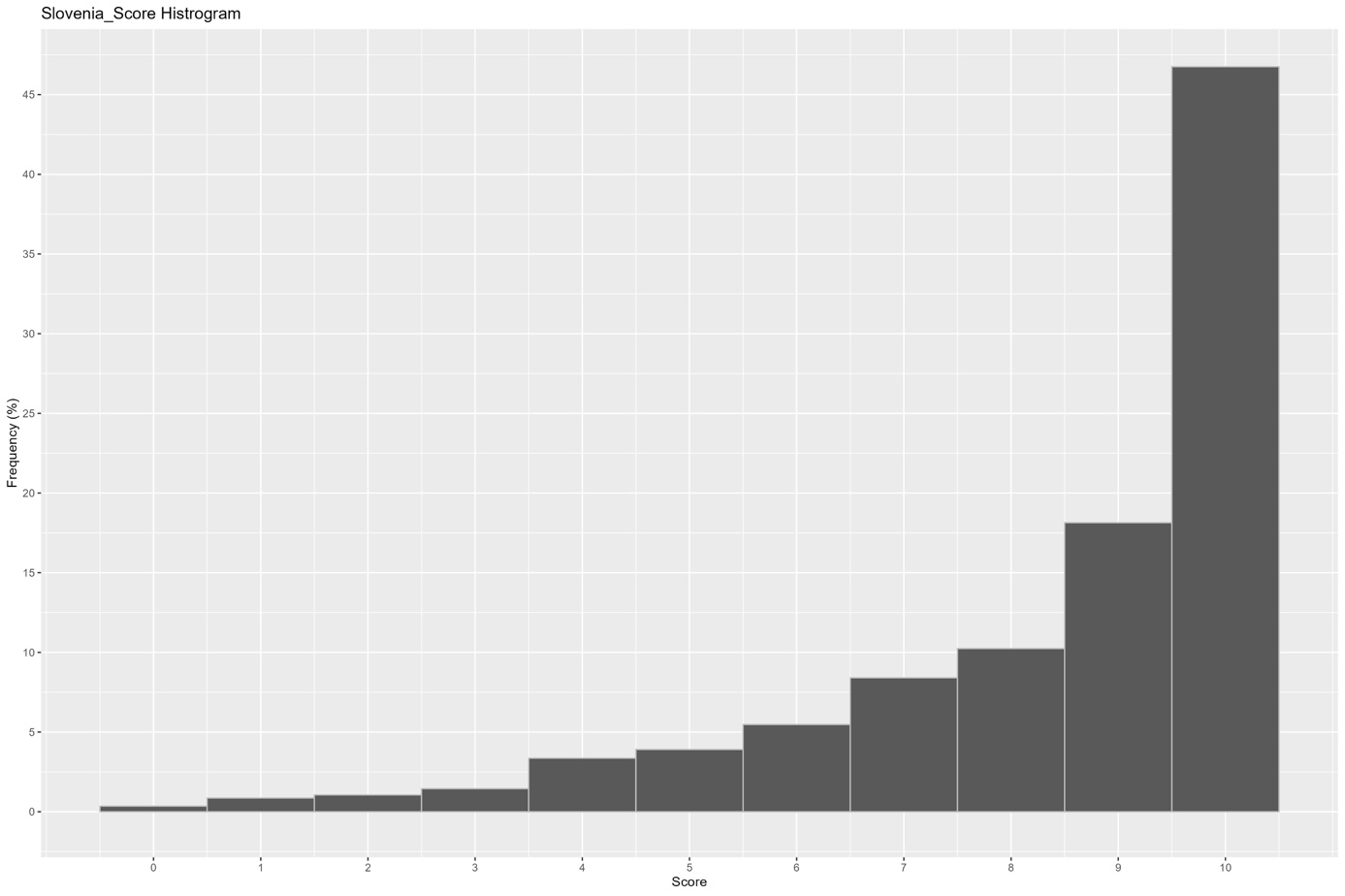

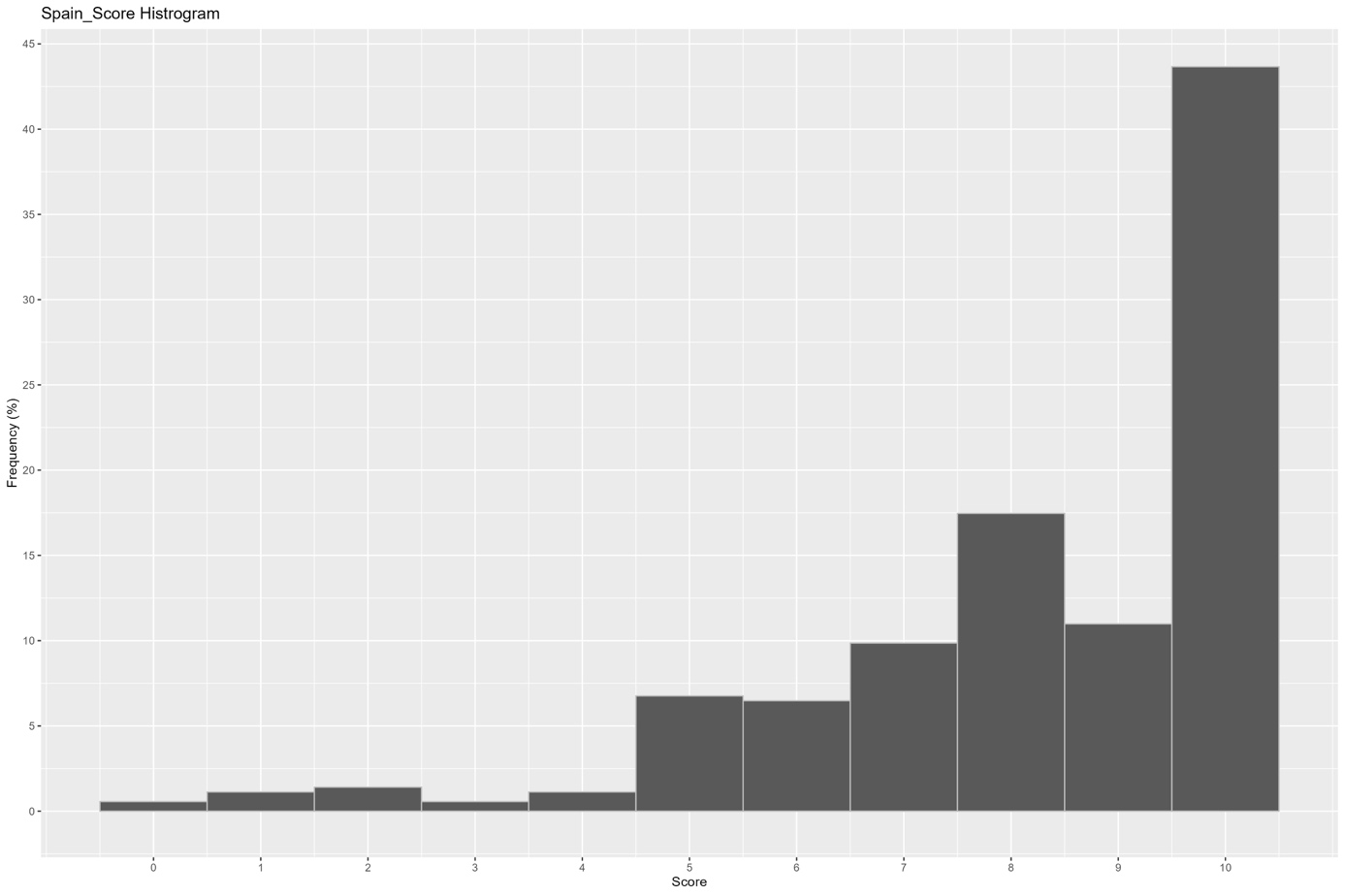

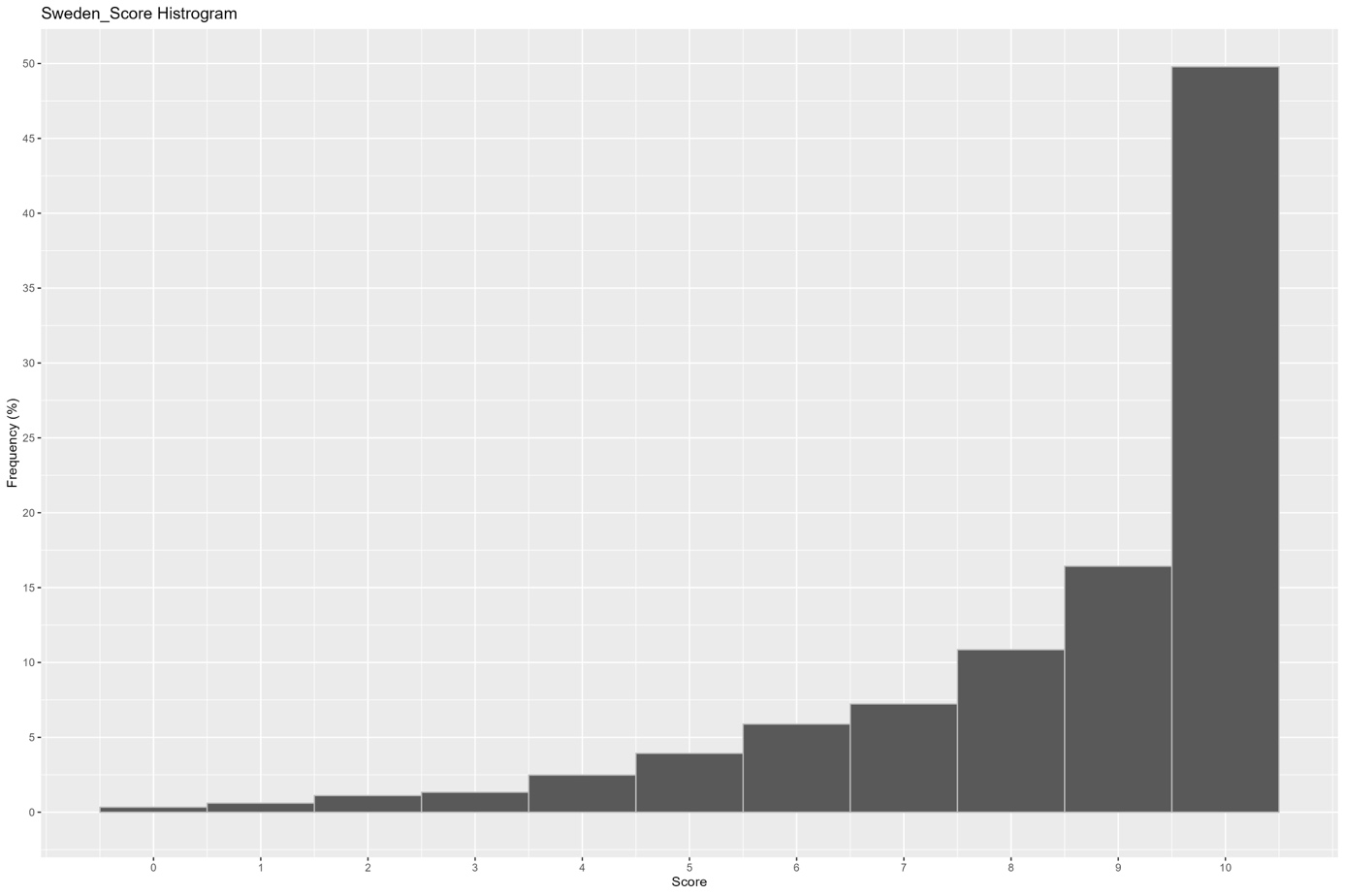

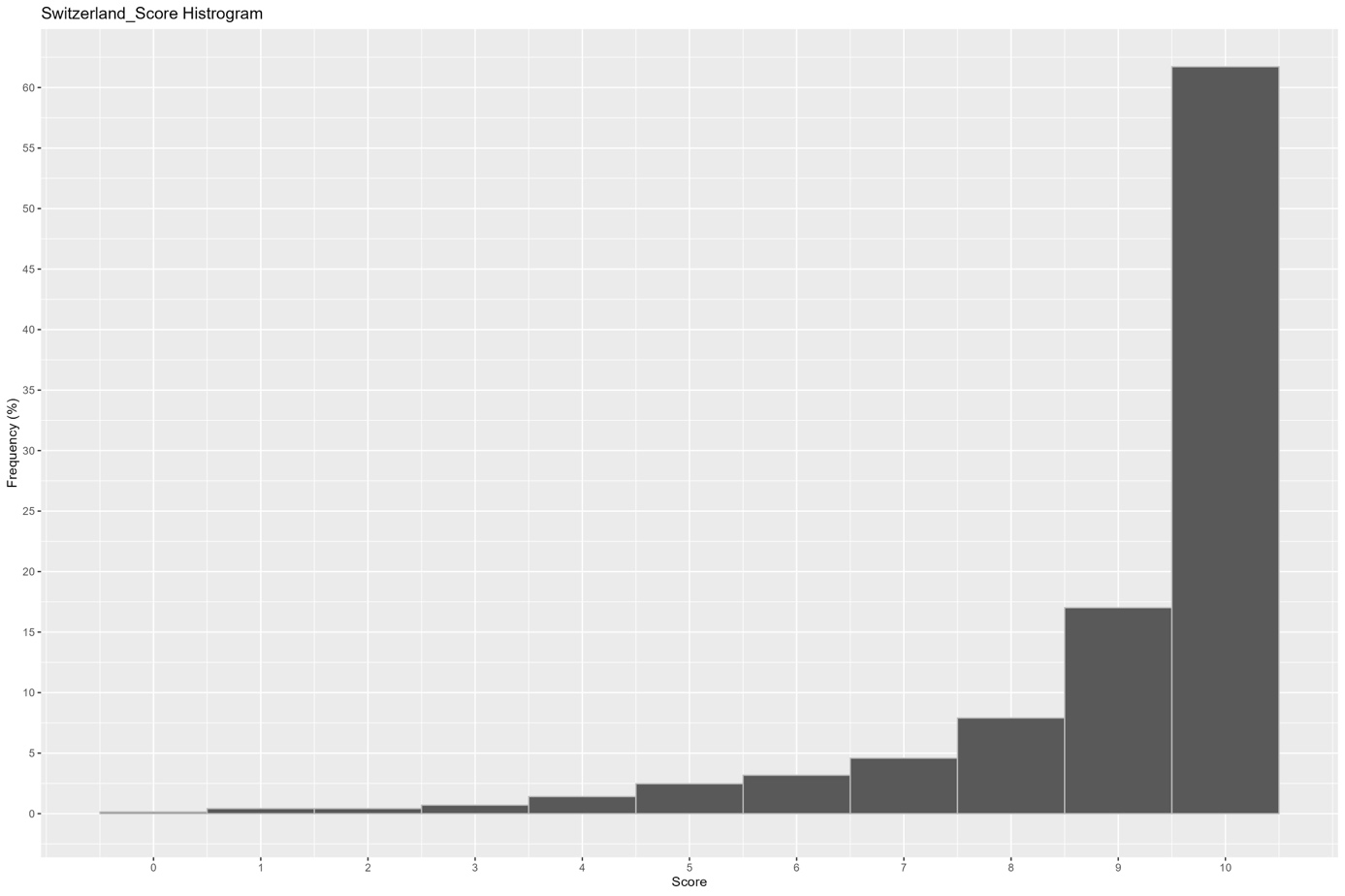
**

Supplement: Supplementary file 2 — File S2. [file BIRT-52-677-s002.docx]
