# Supplementary material for: The Association Between Women's Perception of Birth During the Pandemic, Companion of Choice and Support From Health Professionals: A Cross‐Sectional Study in 20 Countries in the WHO European Region
Source: Birth. 2025 Apr 4;52(4):677–89. doi: 10.1111/birt.12915 (PMC12612363; doi:10.1111/birt.12915)
Supplement: Supplementary file 3 — File S3. [file BIRT-52-677-s003.docx]

| **Supplementary file 3: Sensitivity analysis - Linear model results on the subset of women with a score <10 (n=27,121)** | | | | | | |
| --- | --- | --- | --- | --- | --- | --- |
|  |  |  |  |  |  |  |
|  |  | **Positive birth perception  (composite outcome)** | | | |  |
|  |  |  | **beta coefficient** | **95% CI** | **p-value** |  |
|  |  | **Companion of choice allowed** | | | |  |
|  |  | Always | 0.43 | 0.40; 0.45 | <0.001 |  |
|  |  | Sometimes | 0.23 | 0.20; 0.26 | <0.001 |  |
|  |  | Never | ref | ref | ref |  |
|  |  | **Adequate number of health professionals** | | | |  |
|  |  | Always | 0.34 | 0.31; 0.38 | <0.001 |  |
|  |  | Sometimes | 0.08 | 0.05; 0.11 | 0.010 |  |
|  |  | Never | ref | ref |  |  |
|  |  | **Adequate assistance by health professionals** | | | |  |
|  |  | Always | 0.88 | 0.84; 0.92 | <0.001 |  |
|  |  | Sometimes | 0.53 | 0.50; 0.57 | <0.001 |  |
|  |  | Never | ref | ref |  |  |
|  |  | **Immediate attention by health professionals** | | | |  |
|  |  | Always | 3.52 | 3.48; 3.56 | <0.001 |  |
|  |  | Sometimes | 2.13 | 2.09; 2.16 | <0.001 |  |
|  |  | Never | ref | ref |  |  |
|  |  | **By birth mode and birth interventions** | | | |  |
|  |  | Vaginal birth | ref | ref |  |  |
|  |  | Vaginal birth with episiotomy | -0.30 | -0.33; -0.27 | <0.001 |  |
|  |  | Instrumental vaginal birth | -0.35 | -0.40; -0.29 | <0.001 |  |
|  |  | Instrumental vaginal birth with fundal pressure | -0.63 | -0.68; -0.58 | <0.001 |  |
|  |  | Planned cesarean section | -0.05 | -0.09; -0.01 | 0.161 |  |
|  |  | Cesarean section before the onset of labour | -0.37 | -0.41; -0.32 | <0.001 |  |
|  |  | Cesarean section after the onset of labour | -0.61 | -0.64; -0.57 | <0.001 |  |
|  |  | **Age (years)** | | | |  |
|  |  | 18-24 | -0.13 | -0.18; -0.08 | 0.010 |  |
|  |  | 25-30 | -0.06 | -0.09; -0.04 | 0.012 |  |
|  |  | 31-35 | ref | ref |  |  |
|  |  | 36-39 | -0.02 | -0.05; 0.01 | 0.515 |  |
|  |  | > 40 | -0.12 | -0.17; -0.06 | 0.030 |  |
|  |  | **Type of hospital** | | | |  |
|  |  | Public hospital | ref | ref |  |  |
|  |  | Private hospital/clinic | 0.04 | -0.01; 0.08 | 0.389 |  |
|  |  | **Parity** | | | |  |
|  |  | 1 | ref | ref |  |  |
|  |  | >1 | 0.08 | 0.06; 0.11 | <0.001 |  |
|  |  | **Woman gave birth in the country she herself was born in** | | | |  |
|  |  | Yes | ref | ref |  |  |
|  |  | No | -0.12 | -0.16; -0.08 | 0.004 |  |
|  |  | **Level of education** | | | |  |
|  |  | Junior high school or lower | -0.10 | -0.15; -0.05 | 0.054 |  |
|  |  | High school | -0.14 | -0.17; -0.11 | <0.001 |  |
|  |  | University degree | ref | ref |  |  |
|  |  | Post graduate/ master/ doctorate | -0.05 | -0.08; -0.02 | 0.060 |  |
|  |  | **Country** | | | |  |
|  |  | Other | 0.15 | 0.05; 0.25 | 0.136 |  |
|  |  | Austria | 0.03 | -0.09; 0.15 | 0.815 |  |
|  |  | Bosnia and Herzegovina | -0.61 | -0.70; -0.53 | <0.001 |  |
|  |  | Croatia | -0.34 | -0.39; -0.30 | <0.001 |  |
|  |  | France | 0.33 | 0.25; 0.40 | <0.001 |  |
|  |  | Germany | 0.23 | 0.15; 0.30 | 0.002 |  |
|  |  | Greece | -0.05 | -0.12; 0.01 | 0.403 |  |
|  |  | Latvia | -0.47 | -0.52; -0.42 | <0.001 |  |
|  |  | Lithuania | -0.28 | -0.35; -0.21 | <0.001 |  |
|  |  | Luxembourg | 0.40 | 0.28; 0.53 | <0.001 |  |
|  |  | Norway | 0.68 | 0.64; 0.73 | <0.001 |  |
|  |  | Poland | 0.10 | 0.04; 0.16 | 0.085 |  |
|  |  | Portugal | -0.41 | -0.48; -0.34 | <0.001 |  |
|  |  | Romania | -0.12 | -0.18; -0.05 | 0.083 |  |
|  |  | Serbia | -0.73 | -0.79; -0.66 | <0.001 |  |
|  |  | Slovenia | 0.27 | 0.21; 0.32 | <0.001 |  |
|  |  | Spain | -0.58 | -0.71; -0.45 | <0.001 |  |
|  |  | Sweden | 0.55 | 0.51; 0.59 | <0.001 |  |
|  |  | Switzerland | 0.42 | 0.34; 0.50 | <0.001 |  |
|  |  | Italy | ref | ref | ref |  |
